# Aging rate indicators and the search for anti-aging drugs

**DOI:** 10.3389/fsci.2026.1821393

**Published:** 2026-09-16

**Authors:** Steven N. Austad, Matt Kaeberlein, Richard A. Miller

**Affiliations:** 1 Department of Biology, University of Alabama at Birmingham, Birmingham, AL, United States,; 2 Optispan, Inc., Seattle, WA, United States,; 3 Dog Aging Institute, Boston, MA, United States,; 4 Department of Pathology and Geriatrics Center, University of Michigan, Ann Arbor, MI, United States

**Keywords:** aging, aging rate indicator, anti-aging, drug screening, lifespan, longevity, machine learning, rapamycin

## Abstract

Evidence that drugs, diets, and single-gene mutations can slow aging and extend the healthy lifespan of mammals has begun to convert biogerontology from an observational science into one based on interventions that could provide people with additional years of healthy, productive life. However, the research and development of anti-aging drugs is hampered by the lack of instantaneous and continuous measures of the rate of aging itself. This article presents recent work on anti-aging drugs that are effective in mice, provides evidence for candidate “aging rate indicators” (ARIs), and outlines a roadmap for translating these into clinical research to slow aging in humans. Thus far, 14 agents or combinations have significantly increased the lifespan of mice in studies conducted under the Interventions Testing Program (ITP) of the National Institute on Aging, often when initiated in late adulthood. Recent evidence suggests that many, perhaps all, interventions that slow aging in mice induce common, shared changes in physiological status and molecular pathways. We have proposed 12 such mechanisms as candidate ARIs, i.e., measurable outcomes that reflect a slow-aging state and that could discriminate between normal mice (and perhaps humans) and those exposed to effective anti-aging interventions—thereby accelerating research. Research priorities now include testing the robustness of these ARIs, extending them to include plasma constituents, elucidating the mechanisms through which diverse interventions alter them, uncovering the links between the ARIs and late-life diseases, and extending research to dogs, non-human primates, and humans. The growing recognition of the legitimacy and promise of evidence-based interventions resulting from aging biology should elevate this field to a level of urgency and respect comparable to that of current research on individual age-related conditions.

## Introduction

The pace of aging can be slowed, in mice, by at least three dietary regimens (1–3), at least five single-gene mutations (4–8), and at least eight drugs added to food (9–14). Previous attention to the symptoms of aging, the “hallmarks” of aging, and the supposed “causes” of aging can now be replaced by a focus on a more important problem: identifying the mechanisms that control the rate of aging and using these insights to modulate aging rates. The availability of drugs, diets, and single-gene mutations that can slow aging and extend the healthy lifespan of mammals has begun to convert biogerontology from an observational science into one based on interventions that could help people enjoy additional years of healthy, productive life.

This article presents recent work on anti-aging drugs that are effective in mice and shows how these agents can be used to discover common mechanisms shared by mice in a slow-aging state. We discuss how these shared mechanisms can serve as “aging rate indicators” (ARIs), and we offer a roadmap for translating these advances into the arena of clinical research designed to slow aging in humans.

## Lifespan expansion in mice

It has long been known that lifespan can be extended and late-life diseases postponed by very low-calorie diets and diets deficient in specific amino acids. Single-gene mutations that slow aging in mice were first documented in 1996. More recent studies have discovered at least eight drugs that extend mouse lifespan by 12% or more. This collection of slow-aging mouse models underpins new efforts to find common, shared mechanisms responsible for extending healthy lifespan.

### Diet

The widely held intuition that aging cannot be delayed or decelerated was first refuted in 1935 by the discovery that rodents given approximately 30% less food than they would ordinarily eat live longer (15). By the mid-1980s, research in mice and rats had established the key facts—reviewed in (1, 16)—that calorie restriction (CR): (i) works to extend the lifespan of a very wide range of genetically distinct mouse and rat stocks; (ii) has strong effects in both sexes; (iii) delays most forms of late-life decline, including many lethal and non-lethal illnesses (17), along with declines in fitness; and (iv) leads to concomitant declines in body mass, so that the caloric intake per gram of lean body mass is similar to that of control animals (18). Reducing food intake relative to either a dietary standard or *ad libitum* feeding has been known for decades to extend the healthy lives of multiple vertebrate and invertebrate species (1, 19–22). Mice and rats subject to CR: (i) remain physically active longer; (ii) preserve muscle, heart, and kidney function longer; (iii) show delayed tumor onset (20, 23, 24); and (iv) are more resistant to many toxins and stressors than fully fed animals (25–27). Rodent studies have also identified some harmful effects of CR, typically associated with impaired immune function (20, 28, 29). Although reducing protein relative to carbohydrates without restricting calories also extended life in both mice and rats (30–33), the effect was not as robust as simply reducing calorie intake. These CR diets lead to dramatic changes in cell biology and physiology in nearly all tissues and organ systems, making it difficult to draw convincing, integrated models that link CR to delayed aging and increased lifespan.

Whether the effects of a CR regimen would extend to humans remains unclear. Two long-term studies with rhesus macaques led to inconsistent conclusions. In a study where the control dietary regimen led to higher body weight, restricted feeding significantly increased longevity (34). In a second study where the control regimen resulted in a lower body weight, and the age at which animals started CR was varied, restriction failed to extend life (35). Both studies identified some health advantages (36). A 2-year controlled human study established that people are generally not voluntarily capable of the degree of CR imposed in laboratory research but found cardiometabolic health benefits even from modest CR (37). Notably, however, this study excluded people who exercised regularly to at least the level recommended by the Physical Activity Guidelines for Americans issued by the United States Department of Health and Human Services. More extreme CR, closer to the rodent paradigm, has been practiced by self-selected groups of people for varying but considerably longer intervals, with few obvious health benefits compared with people who achieved a similar level of leanness due to other lifestyle factors (38). These people did, however, show clear benefits when compared with a sedentary, overweight population (39). Thus, CR can clearly slow aging and extend healthspan under highly controlled laboratory conditions, but it is unlikely to be useful to people other than the very small subset able to adhere to an extremely low caloric intake for years or decades.

Diets low in the amino acid methionine also extend the lifespan of rodents, despite normal caloric intake (2, 40, 41). The effect is not attributed to slower development, because lifespan extension occurs even when the intervention is initiated in fully developed 12-month-old mice (42). The recent discovery that diets low in isoleucine also extend rodent lifespan (43) and the earlier observation of lifespan extension in rats given limited amounts of tryptophan (44) suggest that the effects of amino acid restriction on aging may depend on the modulation of protein synthesis.

### Single-gene mutations

The discovery of single-gene mutations that extend the lifespan of the nematode *Caenorhabditis elegans* (45, 46) prompted the prediction (47) that anti-aging mutations would be limited to this species alone owing to its specialized (“dauer”) adaptation for dealing with temporary food shortages. Thus, the 1996 discovery that the Ames dwarf mutation (4)—a mutation in the *Prop1* gene needed for the embryonic development of the pituitary gland—conferred a >40% lifespan extension in homozygous mutant mice was an important breakthrough, giving the first compelling evidence that a single-gene mutation could slow aging in a mammal. Many mutations that interfere with the production of, or sensitivity to, growth hormone (GH) and its mediator, insulin-like growth factor 1 (IGF-1), also extend the lifespan of mice, typically by 20–40%. A large proportion of age-dependent pathologies are delayed in such mutant mouse stocks (48, 49), implying that the extension of lifespan is accompanied by, and very likely caused by, a parallel slowing of conditions that can contribute to ill health and death.

A full list of mutations claimed to extend the lifespan of mice is beyond the scope of this review and would require careful consideration of the quality of the supporting evidence (e.g., Was the work replicated? Were the control mice particularly short-lived? Does the mutation appear to work on more than one genetic background? Does it also slow age effects on non-lethal pathologies and on measures of physical or cognitive fitness?). Nonetheless, a few important themes deserve emphasis. First, many mutations that impair production of or response to GH produce large and reproducible lifespan increases on a variety of background stocks, including Ames and Snell dwarf mice, GH receptor knockouts (GHRKO), and mice lacking either growth hormone-releasing hormone (GHRH) or a functional GHRH receptor (*Ghrhr*) (4, 6, 7, 50, 51). The potential role of IGF-1, a major mediator of GH action, is still unclear. Tissue levels of free IGF-1 are potentially subject to regulation by the enzyme pregnancy-associated plasma protein A (PAPP-A), which can release IGF-1 from some, but not all, IGF-1 binding proteins. Loss of PAPP-A leads to lifespan extension and postponement of many forms of age-dependent pathology (5, 52), possibly reflecting retention of IGF-1 by uncleaved binding proteins and thus diminution of free IGF-1 in one or more unknown tissues. The longer lifespan of PAPP-A knockout mice, despite their normal levels of plasma GH, implies an important role of IGF-1 in mediating the beneficial effects of GH deficiency mutations. Conversely, the absence of lifespan benefits in mice that lack GHR only in the liver (53, 54) argues against a key role for IGF-1 in lifespan determination.

Bartke and colleagues showed that some of the beneficial effects of low-GH mutations are mediated by responses in very young (i.e., prepubertal) animals. In the key experiment, transient (6-week), early-life GH exposure abrogated the lifespan increase seen in saline-injected Ames mice and blocked the multiplex stress resistance typical of fibroblast cultures derived from the skin of Ames mice (55). Early-life GH treatment also blocked the age-related inflammatory changes seen in normal control mice (but not in Ames mice) (56, 57) even though, when the mice were tested at 18 months, they had not been exposed to GH at any point after age 8 weeks.

These and other studies show that the lifespan of mammals can be dramatically extended by mutations at single genetic loci, suggesting that some effects depend on endocrine signals between birth and puberty that exert enduring effects on survival and late-life inflammatory states.

### Drugs

Pioneering studies over the last 20 years provide firm evidence that a variety of drugs can extend lifespan in mice, with accumulating data pointing to delays in multiple pathological and physiological traits associated with aging.

#### Lifespan extension

While numerous studies have reported lifespan extension in mice induced by various agents, many of these studies have methodological weaknesses (e.g., the use of a single isogenic mouse stock, short-lived controls, or controls prone to specific early-life lethal illnesses), and few of these have been replicated by a second group or in a second cohort. Here we focus on work done under the auspices of the United States National Institute on Aging (NIA)-supported mouse Interventions Testing Program (ITP), which aims to standardize research (12). Key features of the ITP protocol include: (i) the use of a genetically heterogeneous mouse stock (UM-HET3) to reduce the likelihood that positive or negative results reflect strain-specific idiosyncrasies; (ii) the replication of each study at three independent sites to determine if positive results are dependent on site-specific environmental variables; (iii) the use of male and female mice; (iv) the use of a sufficient number of mice to achieve 80% power to detect a 10% lifespan effect, even if one site fails to produce data in a given year; (v) the annual public solicitation of suggestions for agents to test; (vi) the publication of all lifespan data, positive, negative, or neutral, for each tested intervention; and (vii) the creation of a biorepository (since the 2015 cohort) of tissues derived from 22-month-old mice to allow for studies of ITP drug-treated mice by researchers at other institutions.

Fourteen agents or combinations have produced a statistically significant increase in lifespan in one or both sexes in ITP studies (Table 1). Seven individual agents, plus the combination of rapamycin and acarbose, led to an increase of 12% or more in median lifespan. Rapamycin had the largest effect of any single agent, leading to a 23% increase in the median in males and a 26% increase in females. The combination of rapamycin plus acarbose led to a 29% increase in lifespan in males, significantly higher than that produced by the same dose of rapamycin alone; this was also the highest percentage increase versus cohort-matched controls of any pharmacological intervention. Rapamycin plus metformin also extended lifespan in both sexes; however, the increase over controls was not significantly higher than that of rapamycin used alone in earlier ITP datasets (60).

Because the log-rank test relies on the useful oversimplification that inter-group hazard ratios are constant across the lifespan and because median survival ages can reflect early-life diseases and risks whose connections to aging are uncertain, the ITP also reports an index of late-life effects (68), based on the Fisher’s exact test statistic for the 90th percentile survival age within the joint distribution of control mice and those in each group of treated mice. This test is more informative than the traditional one, the maximum recorded lifespan in a group, which has poor inter-sample replication and is heavily dependent on population size (69). Six of the tested interventions—rapamycin, acarbose, and glycine for both sexes, and 17α-estradiol, canagliflozin, and 16-hydroxyestradiol in males—met this criterion at *p*<0.05, as did the combinations of rapamycin plus acarbose and rapamycin plus metformin.

At the outset of the ITP initiative, the default assumption was that drugs found to be effective in one sex would be effective in both, because the majority of harmful effects of aging are seen in both males and females. In addition, the effect of CR on lifespan was similar between the sexes. It was therefore surprising to discover that 9 of the 14 beneficial interventions led to lifespan extension in males only (at the tested doses and age ranges), and one other, acarbose, produced consistently larger effects on lifespan in males than in females. Other than rapamycin, only two agents tested to date had significant effects in female mice (captopril) or in both sexes (glycine), and these effects, while statistically significant by log-rank test, were small (i.e., <5% over the control median). The reason(s) for this sex-specific disparity are not clear, representing an important gap in our understanding. Male-specific benefits of canagliflozin may reflect sex- and age-specific variation in drug pharmacodynamics: blood canagliflozin levels in aged females were found to be approximately 3 times higher than those in aged males and approximately 20 times higher than those in young males (11, 63). Canagliflozin administered to females throughout adult life did not significantly alter lifespan in either direction, but canagliflozin initiated at 16 months of age significantly shortened female lifespan while increasing male lifespan (63). This suggests that high blood levels of canagliflozin in aged females may produce toxic effects not seen in males, and may mask any hypothetical positive effects that might otherwise affect females. This idea is now being examined by exposing female mice to canagliflozin at lower doses or only at earlier ages. It is also possible that some fraction of the beneficial effects induced by canagliflozin depend on male-specific factors, such as hormonal or enzymatic traits that distinguish male from female mice. Elucidating why so many of the ITP-tested drugs appear to be male-specific and fashioning better strategies to identify agents that work in both sexes or in females only are future research priorities.

Another surprising result from the ITP is that the benefits of the majority of anti-aging drugs are apparent even when drug exposure is delayed until late middle age (typically 16–20 months). Previously, the default assumption was that substantial lifespan extension would require treatment throughout adult life, because it was assumed that many signs and symptoms of aging were attributable to damage (e.g., somatic mutations, cell senescence or death, or protein cross-links) accumulated progressively throughout adult life (or even from a younger age). In contrast, ITP data showed that essentially complete survival benefits were seen after midlife initiation of rapamycin (9, 58). These unexpected results have implications for human preventive medicine—suggesting that therapy could be effective even when initiated in middle-aged adults. These results also imply that age-dependent changes that contribute to mortality risk are still fully or partially drug sensitive, even in mice that have lived more than half of the median lifespan. Transient exposure to rapamycin early in life (70) can also extend mouse survival, even if these mice are not exposed to rapamycin at older ages. It would be very informative to determine whether the changes induced by very early rapamycin treatment overlap with those that extend lifespan when this drug is administered to adult or aged mice.

#### Age-dependent traits

The majority of laboratory-adapted mice, including approximately 80% of UM-HET3 mice, die as a result of neoplastic disease (71). Therefore, it is important to consider whether survival advantages result from a mode of cancer prevention rather than a more general delay or deceleration of aging processes. If cancer prevention were the only beneficial effect of these drugs, one would expect a reduced tumor burden while other age-associated functional declines and molecular changes across various tissues and organs would remain unaltered. In the case of CR, this possibility was definitively excluded by the late 1990s, as evidenced by the delay of dozens of age-dependent phenotypes in addition to reduced cancer (1).

In drug-treated mice, the strongest evidence to date on this topic comes from the histopathological analysis of rapamycin-treated mice euthanized at 22 months of age (i.e., at an age where >80% of the mice have not yet died) (72). These cross-sectional midlife necropsy protocols are not intended to provide information about the cause of death, but rather to provide information about the effects of drugs on age-sensitive lesions in multiple organs, including those that rarely lead to lethal illness. Rapamycin lowered the incidence of 9 of the 10 age-sensitive changes for which age effects were significant at 22 months of age; the difference in lesion incidence was significant for 5 of the 9 drug-sensitive changes, even in a study with modest statistical power (72). These significant changes were noted in the liver, adrenal glands, endometrium, heart, and tendons. A separate study examined midlife pathology in mice treated with canagliflozin, which extends the lifespan of male mice only. This analysis found canagliflozin-dependent inhibition of lesions in the adrenal glands, heart, blood vessels, liver, and kidneys of male mice only (64), implying that the unknown sexually dimorphic factors responsible for male-specific survival also produce male-specific delays in age-dependent tissue pathology. The inhibition of age-dependent changes in multiple tissues supports the idea that the drugs in question retard aging and not merely age-dependent lethal illnesses such as neoplasia. These findings are consistent with several studies on rapamycin in UM-HET3 and C57BL/6 mice, indicating that rapamycin delays age-related decline in cognitive function (73, 74), the kidneys (75), the tendons (76), the ovaries (77), hearing (78), and the intestines (79, 80). Rapamycin treatment in midlife is also sufficient to at least partially reverse functional declines and molecular changes in the heart (81, 82), the immune system (83), and the oral cavity (84).

There is also a small but growing body of evidence on whether drugs that extend mouse lifespan also slow age-related changes in tests of physiological fitness. Rapamycin, for example, diminished the age-dependent declines in spontaneous in-cage activity at two of the three ITP sites (58); for unknown reasons, age did not appear to have any effect on activity at the third site. Hypothalamic inflammation, which is inhibited in several genetic models of slower aging, is also delayed, in males only, after treatment with either acarbose or 17α-estradiol (85), each of which has a preferential survival benefit in males. Acarbose also produces a male-specific inhibition of several indices of cardiac aging in UM-HET3 mice, including left ventricular mass and isovolumetric relaxation time (an index of diastolic function) (86)—both of which are also characteristic of aging in humans. In both sexes, acarbose opposes age-related changes in grip strength and rotarod performance, a measure of strength and balance (86, 87). Similarly, 17α-estradiol inhibits the effects of age on grip strength and rotarod performance, even when started in late middle age (88), and in a sex-specific way. All these studies support the hypothesis that drugs that extend lifespan also delay the effects of aging on multiple forms of pathological and physiological change; however, the evidence is inconclusive, with many tissues, traits, and fitness tests still unevaluated. More extensive data on cognitive tests in mice exposed to a variety of lifespan-extending drugs would be particularly valuable.

## Aging rate indicators

A physiological or biochemical trait that is shared by all varieties of slow-aging mice—an “aging rate indicator”—could speed the search for anti-aging drugs and provide new insights into the biology of aging and late-life illnesses.

### Shared mechanisms regulating aging rates

Diets, drugs, and mutations that extend mouse lifespan by postponing late-life lethal diseases appear, *prima facie*, to work through a wide range of primary targets. Some of these modulate GH/IGF-1-dependent pathways, some modulate glucose peak levels, some inhibit mammalian target of rapamycin (mTOR) complex 1 (mTORC1), some restrict specific classes of nutrients, and some (e.g., 17α-estradiol and 16-hydroxyestradiol) presumably affect steroid-sensitive cells in one or more unidentified cell types. Surveys of messenger RNA (mRNA) changes induced in the tissues of many of these mice suggest that each intervention leads to its own particular set of transcriptional changes, with a limited amount of overlap (89), and proteomic analyses also provide little to no evidence of shared changes in the tissue proteome between different anti-aging interventions in mice (90).

Despite these grounds for pessimism, recent evidence suggests that many, perhaps all, interventions that slow aging in mice induce a set of common, shared changes in physiological status and molecular pathways. Initial studies on 10 varieties of slow-aging mice (reviewed in 91) compared CR mice, five single-gene mutants, and four kinds of drug-treated mice to their respective controls and noted many common features (Table 2). These included: (i) increased levels of the mitochondrial uncoupling protein 1 (UCP1) in brown fat, subcutaneous white fat, and intra-abdominal white fat (93, 96, 102); (ii) changes in adipose tissue macrophages that diminish the production of inflammatory cytokines (93, 102); (iii) increased production of fibronectin type III domain-containing protein 5 (FNDC5), a muscle protein whose cleavage product irisin may be responsible for the beneficial changes in fat (93, 96); (iv) increased levels of glycosylphosphatidylinositol-specific phospholipase D1 (GPLD1), a liver enzyme thought to stimulate improved cognitive abilities after exercise (94, 95, 103); and (v) increased levels of two proteins—brain-derived neurotrophic factor (BDNF) and doublecortin (DCX)—thought to protect brain cells against damage and support production of new neurons (57, 94, 95). Other shared features include changes in intracellular control pathways, namely: (i) diminished mTORC1 activity in the liver, heart, muscles, and kidneys (54, 97, 101); (ii) diminished protein kinase activity in the mitogen-activated protein kinase (MEK)/extracellular signal-regulated kinase (ERK) pathway, which controls translation (97, 98); (iii) diminished activity in the MEK3/p38 protein kinase pathway that controls the production of acute-phase proteins after inflammation (97, 98); and (iv) increased levels of proteins regulated by the selective translation of mRNA (“cap-independent translation”; CIT) of specific proteins, of which GPLD1 may be a key member (95, 99). A more recent study also showed similar changes in the mTOR and MEK pathways in isoleucine-restricted mice (3). Changes in pro-inflammatory signals mediated by NF-κB were noted in the majority, but not all, of the tested models (92).

Notably, the changes in these pathways observed in the long-lived Ames dwarf mutant can be permanently blocked by transient exposure of newborn Ames weanlings to GH injections for a 6-week period, a regimen that also blocks lifespan extension, decreases cellular stress resistance, and blocks resistance to hypothalamic inflammatory changes characteristic of Ames dwarf mice and other long-lived mice (57, 104). This suggests that the early-life epigenetic changes that mediate the effects of varying growth hormone levels on aging rates and lifespan also alter levels of these shared common factors, which can therefore be classified as ARIs.

### Candidate ARIs

These cellular and molecular features, shared across at least 10 varieties of slow-aging mice—with supportive evidence now emerging from an eleventh slow-aging model based on isoleucine restriction (3)—suggest that they may be indicators of a slow-aging state that can be induced by a wide range of genetic and non-genetic interventions. The underlying concept is that the rate of aging (i.e., the timing with which aging processes lead to signs and symptoms of aging) can be monitored by measurements of surrogate traits, or ARIs, such as those that discriminate between normal and slow-aging groups of mice. The rate of aging varies across species (e.g., dogs age more slowly than mice but more rapidly than humans) and across breeds within species (e.g., small dogs and horses versus larger breeds) (3, 105, 106). We propose, as a testable framework, that ARIs might be useful in distinguishing normal mice (and perhaps humans) from those who have been exposed to an intervention that slows aging.

Importantly, ARIs are fundamentally different from the more familiar “biomarkers of aging.” Biomarkers of aging are conceptualized as traits that change with age, at least in adult life, and can thus provide information about how old an individual might be. For example, a 60-year-old person who resembles an average 70-year-old in many ways (e.g., changes in vision, hearing, skin elasticity, immune response, cardiovascular reserve, DNA methylation patterns, and mRNA levels in peripheral blood cells) might be considered biologically older than someone of the same chronological age who more closely resembles a 50-year-old in the majority of these traits. Metaphorically, a collection of biomarkers of aging, or a summary statistic derived from them (e.g., “biological age”), is similar to a car’s odometer, which indicates how many miles the car has been driven. To use a collection of biomarkers to study a candidate anti-aging intervention requires at least two measurements, separated by an interval sufficient to allow for age-related changes to occur in the control group and to occur at a hypothetically slower rate in the treated group. Since many age-sensitive traits show wide variation within a test population and change fairly slowly with age, using traditional biomarkers of aging to evaluate an anti-aging intervention will probably require a large population size and take many years for humans. In this analogy, ARIs are similar to a car’s speedometer, which instantaneously estimates the rate of change, namely, a car’s current speed or an individual’s current rate of aging. ARIs provide no information about a subject’s biological age, in the same sense that a speedometer does not discriminate between new and old cars. In principle, however, ARI measurements can provide information about aging rates without the need to wait for the test subject to become older. In other words, ARIs can be measured once, at a single age, in young or middle-aged animals, to provide information about aging rates, unlike the more familiar biomarkers of aging, which must be measured at least twice, at two ages.

Figure 1 shows the hypothetical effects of an anti-aging drug on a biomarker of aging and an ARI. The drug diminishes the slope for the biomarker (A). The biomarker level gradually diverges from that of the untreated control group as aging progresses. In contrast, the ARI (B) rapidly transitions from its normal level (in this hypothetical case, an age-invariant level) to a level (in this case, a higher level) characteristic of the “slow-aging state” and therefore seen in many varieties of slow-aging mice.

In this formulation, the only criterion for a measurement to be considered a candidate ARI is that it be different from controls in multiple, and preferably all, varieties of slow-aging mice. The direction of the effect of age on an ARI is irrelevant—it may increase, decrease, or remain unchanged (as in the hypothetical example in Figure 1). Similarly, information about the levels of candidate ARIs in putative models of “accelerated aging” is not pertinent, since the proposed definition is based entirely on levels in normal versus slow-aging mice. Nor is it claimed that interventions with a large effect on lifespan will show larger changes in ARIs compared with interventions with more modest effects. In the current working conceptual framework, the only claim is that ARIs will be different (higher or lower) in slow-aging mice versus sex- and age-matched controls.

In principle, changes in the level of an ARI, or a collection of ARIs, could greatly accelerate the search for anti-aging drugs. At present, a drug-induced extension of median and maximum lifespan is considered *prima facie* evidence of an anti-aging effect and the first step in showing that the drug in question postpones a wide range of age-dependent changes, including health outcomes important to healthspan. Such a study in mice can cost US$100,000–300,000, depending on the statistical power chosen and the effect size to be evaluated, and requires 3–4 years from birth to reach the 90th or 100th percentile survival age. The majority of the data on changes in ARIs induced by anti-aging drugs were derived from protocols in which mice were exposed to drugs or the CR diet at 4 months of age and then evaluated 8 months later (i.e., at 12 months of age) and required only 10–20 mice of each sex per test group. If such a procedure can be shown to discriminate between effective and ineffective candidate drugs (i.e., to predict the outcome of full-scale lifespan studies), studies could be conducted approximately four times more quickly and at approximately one-twentieth the expense. A similar approach (the DunedinPACE measure) has shown promise in assessing aging rates in human clinical settings (107–109).

## Research roadmap for ARIs and anti-aging drugs

A set of validated ARIs could significantly accelerate research on anti-aging drugs. This section offers a roadmap of priorities for the incorporation of ARIs into clinical research (Figure 2)—including key knowledge gaps relating to ARIs themselves, the pathophysiological implications of the underlying shared anti-aging mechanisms, and the roles of various animal models.

### Knowledge gaps: current goals for ARI studies

The research completed to date leaves many important questions unaddressed, and new studies to clarify these issues will help to determine whether ARI data will live up to their initial potential. We propose the following priorities:

- The majority of the published studies have emerged from a single laboratory group using a single (though genetically heterogeneous) mouse stock. Replication in other animal colonies is needed to test the robustness of the findings and the underlying model.
- The published articles have focused on contrasts between slow-aging mice and their relevant control littermates but have not yet been extended to address possible differences among “normal” mice (i.e., mice that have not been exposed to anti-aging drugs or diets and that do not carry a mutation known to slow aging and extend lifespan). One obvious design is to test ARIs in adult mice whose late-life fitness and age at death would then be measured, to see if those adults with ARIs associated with slow-aging mice do indeed preserve fitness and survive to older ages than others in their cohort. Such a design depends heavily on the development of ARIs that can be measured in plasma, since plasma can be sampled without harming the animals.
- Two published ARIs—irisin and GPLD1—can be measured in plasma samples; however, a more comprehensive set of plasma-based ARIs is needed to understand the extent to which ARIs predict fitness and lifespan among individual mice and to extend the research to human drug testing. Evaluating plasma metabolomic and proteomic datasets to identify features that correlate with ARI measurements in the same individuals will help to address this need.
- It is not yet clear how quickly ARI status changes after exposure of a mouse to an anti-aging drug, whether such changes are abrupt or gradual, or whether the timing or rate of alteration will differ among ARIs. If, for example, some ARIs change more quickly than others after exposure to an anti-aging intervention, these ARIs might be particularly useful for initial rapid screening of candidate interventions.
- Some promising ARIs are themselves altered by age. For example, there is an age-related decline in UCP1 levels in adipocytes (110, 111) and a relative increase in proinflammatory macrophages compared with anti-inflammatory macrophages (112, 113). It is not yet clear whether ARIs that can distinguish normal from slow-aging mice at young adult ages will also serve this function at older ages, although ARI data obtained from the adipocytes, macrophages, livers, muscles, and brains of 15- to 18-month-old Ames dwarf mice imply that ARIs may be detectable in both young and older adults (57).
- The conceptual model presented here is the simplest one, in which changes in the pace of aging affect all age-sensitive cells, tissues, and inter-organ circuits. Thus far, there is only one clear example of an exception to this idea—the failure of rapamycin to elevate UCP1 in white adipose tissue depots (93). Plausibly, some drugs that slow aging may also affect cell or tissue features unrelated to their effects on aging, which may disrupt the linkage between a specific ARI and the rate of aging. Indeed, previous studies had already shown that rapamycin exposure diminishes UCP1 in white adipose tissue (114, 115), presumably through mechanisms different from those by which rapamycin affects aging, lifespan, and all other tested ARIs, including UCP1 in brown adipose tissue. Despite the simplicity of the “rate of aging” model, it is possible that some interventions, or some genetic factors, might lead to slower age-related changes in specific organ systems, such as those related to cognitive decline, immune senescence, or preservation of muscle mass and strength. It is also quite likely that some of the traits that are altered by a wide range of anti-aging drugs are altered by other factors, including drugs and interventions that do not modify the aging process. Physical or mental exercise, for example, might in principle have effects on brain BDNF or muscle FNDC5 that are not part of a broader program of anti-aging effects. A more comprehensive assessment of candidate ARIs, in connection with a broader evaluation of a wider range of age-sensitive functional changes, could help refine the original model by providing evidence of markers of changes in the rate of aging in specific domains.
- One approach to identifying plasma-based ARIs in humans could be to collect data on plasma metabolites or proteins in a set of several hundred healthy adults at an age, perhaps 55 or 60 years old, at which the majority of individuals are likely to survive in good health for at least another decade. A second plasma sample taken after that 10-year interval could be used to evaluate, for each participant, a change score for each metabolite found to be age-sensitive in the group as a whole. The final step could be to determine which metabolites or proteins in the original sample were able to predict the pace of age-related changes in the metabolite profile or in measures of health, physical fitness, and cognitive fitness. These plasma constituents could be excellent candidates for ARIs in humans and could be incorporated into future studies of potential anti-aging drugs.

### Pathophysiological implications of shared anti-aging mechanisms: upstream and downstream challenges

The evidence that ostensibly distinct anti-aging interventions and anti-aging genes all trigger a common set of shared changes presents new opportunities for both pathobiology and biological gerontology.

Pathobiology questions are all “downstream” of the shared mechanisms and focus on the connections between the shared features and the important diseases seen in mice and humans. Many of the current set of ARIs were selected because of their known or suggested links to such diseases, including the link of adipose tissue browning to diabetes and metabolic syndrome, the connection of BDNF and DCX levels to neurodegenerative diseases, and the association of FNDC5/irisin and GPLD1 with the benefits of physical exercise. Evidence that these plausible mediating control pathways are themselves favorably altered by a large group of anti-aging drugs, diets, and genetic mutations offers new tools for research, potentially including clinical prevention trials. For example, data showing that four different anti-aging drugs induce increases in BDNF and DCX levels should prompt new studies to determine if these agents can postpone age-related changes in brain function, either in animal models or humans. The ability of the same drugs to postpone age-related increases in multiple forms of cancer, cardiac disease, and other conditions could provide mechanistic links between brain aging and other adverse consequences of aging. Similarly, the use of nominally “anti-aging” drugs to postpone age-related hematopoietic and parenchymal neoplastic diseases might be of interest to research oncologists, even though these drugs did not emerge from the cancer research community.

Studies that look “upstream” (i.e., those that attempt to identify the unknown pathways that connect the numerous anti-aging interventions to a broad array of tissue changes) are more challenging but likely to be at least as valuable. It is not yet clear how any of the anti-aging drugs, diets, or genetic mutations alter fat tissue, macrophage subset ratios, selective mRNA translation, or the status of muscle, liver, and brain. Each specific intervention could alter the large set of ARIs through unrelated pathways still to be elucidated. It appears to be more likely, however, that the coordinated set of changes seen in many cells and tissues reflects the action of a switch from a normal- to a slow-aging state. The cellular basis for such a switch is unknown and could in theory reflect a specific cell-autonomous alteration in many cell types, such as a change in expression level of one or several proteins. Alternatively, the hypothetical slow-aging state could reflect a change in a specific cell type—perhaps in the central nervous system, the gut, the vascular endothelium, or an endocrine organ—that then influences other cell types throughout the body. Data on the best-studied and clearest examples of slow-aging groups within species (i.e., dwarf mice, CR rodents, and breeds of horses and dogs) do not exclude either of these two ideas. ARIs that discriminate between normal and slow-aging mice, regardless of the specific intervention that triggered the slow-aging state, provide important resources for testing molecular hypotheses about the so-far unknown basis of coordinated changes in ARIs, the pace of aging, and the risk of many late-life forms of illness and decline.

### Artificial intelligence and machine learning approaches

The term “artificial intelligence” (AI) encompasses a diverse range of algorithmic strategies to address problems such as prediction of protein folding, identification of abnormalities in lung X-ray images, extrusion of passable text, tagging of individual crime suspects in surveillance videos, and writing computer code. There is understandable hope that AI methods could be applied to large omics datasets, perhaps combined with the results of clinical and physiological variables, in order to select drugs and nutritional interventions that could slow aging and extend lifespan.

Although the glamour and well-publicized successes of AI algorithms have raised high hopes for their future contribution to the search for anti-aging drugs, some clear obstacles will need to be overcome before this potential can, perhaps, be realized. First, the development of these strategies may well depend on careful attention to the goal of the training exercise. In particular, an algorithm that succeeds at discriminating between older and younger individuals, or at estimating life expectancy in a set of healthy adults, may have little or no relevance to the problem of discriminating slow-aging adults from those aging at average rates. Indeed, some evidence already indicates that omics profiles optimized to track the effects of age on phenotypes in mice may be ineffective at predicting life expectancy. Many such efforts have, so far, been based on transcriptomic data (89, 116), since these datasets are now widely available and relatively inexpensive to produce. However, the often weak and context-dependent correlation between age-dependent changes in mRNA and changes in the corresponding proteins (117–119) is likely to diminish the effectiveness of predictive algorithms based on mRNA patterns alone.

Second, the paucity of well-validated anti-aging drugs poses serious limits on AI-based assessments of omics data in the search for novel candidate drugs, with a sophisticated example provided by a recent study (120). Training AI agents to distinguish photos of dogs from those of cats, for example, begins with the acquisition of many images of each—but very few drugs have been demonstrated to extend mouse lifespan, and none are known to slow aging in humans. Thus, groups wishing to deploy AI methods must rely on poorly validated surrogates, such as lifespan effects seen in invertebrates, effects on pathways suspected to be involved in the aging of one or more tissues or subcellular structures, or correlations with transcript patterns and methylation profiles considered to be plausible indices (“clocks”) related to “biological age.” The strengths and weaknesses of using this heterogeneous collection of aging-adjacent data to identify drugs that extend mouse lifespan are still largely untested; however, it seems probable that careful curation of training data will be required to compensate for the paucity of datasets from validated anti-aging drugs that extend mouse lifespan.

Large language models (LLMs) capable of generating human-like natural language text have also attracted widespread attention from investors and end users and have proven their ability to assist with essay composition, generation of meeting summaries, and production of useful computer code. LLMs often do well when asked a question for which expert consensus exists (e.g., “which drugs can lower blood pressure?”). In areas where expert opinion is conflicted (i.e., “what drugs can extend human lifespan?”), LLMs may be able to provide a summary of current controversies or overconfident consensuses, depending on their training base and predispositions. There is, however, no present evidence that LLMs can surpass the poor performance of authentic experts, whose suggestions of candidate drugs for the ITP lead to lifespan extension less than 20% of the time.

Despite the aforementioned challenges, AI and machine learning (ML) approaches are increasingly being used in aging research to analyze large molecular datasets generated by diverse longevity interventions. The availability of a wide variety of slow-aging mouse models has allowed a search for mRNAs, proteins, or metabolites that are altered in the same direction across many such models; however, the results to date have been somewhat disappointing. An assessment of proteomic data from genetically heterogeneous adult mice exposed to anti-aging interventions (i.e., a CR diet, Snell and GHRKO mutations, and four drugs), the same mice used for the ARI studies summarized in Table 2, did not find any protein that met the authors’ criterion for significance in all of the models (90). A network analysis of this proteomics dataset found altered network properties in mice treated with acarbose, rapamycin, 17α-estradiol, and the CR diet, and a combination of metabolomic and proteomic data from the same mice revealed shared shifts in lipid oxidation patterns (121). A recent study from the Gladyshev research group (120) has asserted that autonomous AI agents can “discover aging interventions from millions of molecular profiles,” although it is not yet clear whether candidate anti-aging drugs identified by this approach will actually extend lifespan and postpone age-related decline in mice or humans.

A recent study (122) illustrates how ML, a specific subset of AI methods, can provide insights into common features of anti-aging models, point toward specific new targets for drug selection, and help the translation from mouse models to human investigations. Plasma metabolomic datasets, containing abundance values for 1,051 metabolic features, were used to train an ML-based regression model using extreme gradient boosting (XGBoost). The plasma samples came from adult UM-HET3 mice that had been exposed, from 4 to 12 months of age, to rapamycin, acarbose, 17α-estradiol, canagliflozin, or the CR diet; or from untreated age-matched controls. Since actual age-at-death data were not available for these 12-month-old mice, the training set assigned each mouse an estimated lifespan corresponding to the percentage increase in median lifespan for mice of the same sex and treatment group. Crucially, each model was trained on data from control mice plus mice in four of the five available slow-aging treatment groups, and then it was used to predict the estimated lifespan increase of mice from the fifth variety of slow-aging mice, none of which had been included in the training process. This “novel intervention test” thus mimics a practical situation in which trained models are intended to estimate the efficacy of drugs for which the actual lifespan effects are not yet known. With group sizes in the range of 10–15 mice per treatment per sex, models trained on data from males predicted lifespan increases in the omitted group with *p*<0.05 compared with control mice for each of the five interventions (see Figure 3). Models trained on plasma peptide datasets instead of metabolite data were equally successful in this task. Additionally, models trained on plasma metabolite data from male mice could also predict longevity extension when applied to plasma samples from female mice, even when the females had been treated with agents (i.e., 17α-estradiol and canagliflozin) that do not extend female lifespan, implying that at least some of the metabolomic changes shared by slow-aging mice are seen in both sexes.

These results are promising but need further expansion. It is unclear if mice treated with drugs that do not extend their lifespan can be cleanly discriminated from those treated with effective anti-aging agents, whether the results seen in plasma samples from 12-month-old mice can be replicated using samples from older or younger animals, or whether the same approach can be equally effective in other stocks of mice or when using other metabolomic pipelines. It is also unclear how long it takes for an effective anti-aging drug to alter metabolite profiles in a direction consistent with a slow-aging state; the data by Badenoch et al. (122) were obtained after an 8-month period of exposure, but shorter periods would be more convenient.

The ML approach also produces a rank-ordered list of the specific metabolites that make the greatest contribution to predictive power, and these lists can provide useful hints about both drug selection and biological patterns implicated in aging rate control. Figure 4, for example, based on the results shown in the study by Badenoch et al. (122), lists the 20 metabolites with the highest predictive power in the plasma of male mice used in the XGBoost model, showing the effects of each of the five interventions individually. Some of these metabolites show a monotonic change, in the sense that they respond in the same direction to each of the five interventions (red arrows in Figure 4). These metabolites show the expected behavior of ARIs, as previously described. Whether metabolites also show consistent changes in other varieties of slow-aging mice remains to be determined. Other metabolites affect the predicted lifespan increase even though they do not show monotonic change. The information captured by metabolites that do not change in the same direction in response to all interventions would be missed by analytical approaches that only consider outcomes that change uniformly in all varieties of slow-aging mice; however, this information contributes to the discriminatory power of non-linear ML algorithms.

If the success of this ML approach proves replicable, it may have important implications. Currently, only approximately 15% of agents approved for testing by the ITP produce significant lifespan benefits at the dose at which they are initially tested, and a comparatively rapid and inexpensive screening process that improves this rate could greatly speed up the identification of new anti-aging drugs for further study in mice and, perhaps, humans. In addition, specific metabolic features with an especially strong impact on predicted group lifespan may suggest new targets or systems for drug discovery. For example, triglycerides (TGs) are significantly overrepresented among the influential plasma metabolites shown in Figure 4. Further inspection revealed that TGs with longer-chain fatty acids were typically increased in abundance in the slow-aging mice, while TGs with short-chain fatty acid components tended to be less abundant in these mice (122). This observation suggests that drugs that modulate fatty acid chain length or the selection of specific fatty acids for TG synthesis could potentially mimic this pattern and perhaps have beneficial health effects. Lastly, patterns of change in the plasma metabolome associated with slow aging in adult mice could provide a bridge to parallel studies of pharmacological effects of candidate anti-aging drugs, or other anti-aging interventions, in humans. If drugs that extend mouse lifespan indeed produce similar sets of metabolomic shifts in human plasma, this would suggest that these might also have anti-aging effects in humans, warranting an especially high priority for clinical and experimental studies.

### Invertebrate models

Simple invertebrate and cell-based organismal models, such as budding yeast *Saccharomyces cerevisiae*, the nematode *Caenorhabditis elegans*, and the fruit fly *Drosophila melanogaster*, served as the workhorses of aging research during the late 1990s and early 2000s, ushering in a new era of molecular, mechanism-based gerontology that increased the credibility of the field within the broader scientific community. One of the most powerful features of these simple models is their ability to perform genetic studies of longevity at an unprecedented scale. This was particularly true in *C. elegans*, where newly developed RNA interference (RNAi) technology could easily be adapted to the study of lifespan. By the turn of the century, two large RNAi libraries had been constructed containing more than 10,000 unique bacterial clones, each expressing a single double-stranded RNA molecule capable of silencing the expression of the corresponding worm gene simply by feeding the animals the clone of interest (123, 124). Two groups, headed by Cynthia Kenyon and Gary Ruvkun, began using these libraries immediately to screen for genes that limited longevity in worms by looking for RNAi clones that increased lifespan when used as a food source. Over the next decade, these and other groups identified dozens of novel longevity-regulating genes spanning multiple functional categories and biological pathways (125–128).

Critically, some aspects of biological aging are highly conserved across the eukaryotic evolutionary tree. Between yeast and worms, organisms separated by more than a billion years of evolution, orthologs of known aging genes in one organism were found to be nearly five times more likely to play a similar role in determining longevity in the other organism (129). Notably, this was shown in the replicative lifespan of yeast, a purely mitotic form of aging, while *C. elegans* are entirely post-mitotic as adults, with the exception of the germline. Many genes that regulate aging in worms and flies are now known to play a similar role in mice, in some cases with correlative evidence in humans. These include mTOR, hypoxia-inducible factor 1 (HIF-1), adenosine monophosphate-activated protein kinase (AMPK), insulin and IGF-1 receptors (along with several downstream components of that signaling cascade), electron transport chain components, and forkhead box O (FOXO) family proteins (130, 131).

The *C. elegans* model has proven utility in screening lifespan-modifying interventions, and worms share with mammals some aspects of biological aging. Therefore *C. elegans* continues to provide an opportunity for high-throughput research to identify longevity drug candidates for validation in longer-lived and more expensive mouse models. Some drugs, including rapamycin (9, 132) and possibly spermidine (133, 134), appear to extend lifespan in both species. In some other cases, agents that extend worm lifespan, such as urolithin A, have produced beneficial effects on age-sensitive health indices in mice (135). Other agents, such as metformin (60, 136), nicotinamide riboside (62, 137), and alpha-ketoglutarate (63, 138), have been reported to extend mouse lifespan in studies that could not be replicated by the ITP consortium. Although some drugs may extend the lifespan of both worms and mammals through autonomous effects exerted on cell biology—rapamycin being the most salient example—others may benefit *C. elegans* by affecting processes that do not play a role in mammalian aging.

Conversely, some drugs that improve mouse lifespan affect tissues, organs, or neuroendocrine pathways that evolved in the vertebrate lineage, and *C. elegans* screens would not be expected to model these mammal-specific effects with much fidelity. Some proven anti-aging drugs, such as acarbose, modulate digestive processes in the gastrointestinal tract of mammals that have no clear parallel in nematodes. Mammals employ insulin, IGFs, and IGF-binding proteins as components of complex, multicellular feedback loops that may not be faithfully mimicked by insulin-like proteins in worms and other invertebrates. Some drugs that show benefits in mice are likely to work by perturbation of cell non-autonomous pathways, for example by alteration of circuits that translate nutrient availability from the gut to the brain, pancreas, and multiple target cells, including the liver and adipocytes. Sex-specific drug responses in mammals may not be well modeled in hermaphroditic worms. The roles of many intercellular signaling pathways, including those of multiple steroids and steroid receptors, may have changed over the last 500 million years of evolution since the Cambrian explosion that produced both invertebrates and the ancestors of mammals. From this perspective, we suspect that broad searches for drugs that extend the lifespan of *C. elegans* may be able to identify drugs that have intrinsic effects on age-sensitive cellular properties. However, such searches may prove less successful in finding drugs that work in mammals, either because they alter intercellular signals, or because they alter molecular pathways that postdate the divergence of mammals and nematodes from their last common ancestor.

The power of worms lies in their extremely rapid aging phenotype and low cost of use. Worms age approximately 50 times faster than mice, and the development of new robotic technologies coupled with AI allows for the determination of worm lifespan at low cost. One ambitious project has proposed to quantitatively assess the impact of one million interventions, single-molecule and combinatorial, in a few years at a cost of less than US$5 million (139). A screen at this scale is likely to lead to many novel discoveries and interventions that might also be worth testing in mice, and, perhaps eventually, in humans. The ability to measure lifespan in hundreds of thousands or even millions of experiments would also afford the opportunity to perform dose–response profiles at scale, something that is not feasible in mice. This approach thus appears quite promising for identifying candidate longevity interventions with a large effect size. However, presently, there are insufficient data to estimate how frequently drugs that increase lifespan in worms will have a similar effect in mice, a problem exacerbated by challenges of replicability for the same drugs between laboratories and mouse strains.

### Marmoset and dog studies

As we move closer to validated ARIs in mice, it is not clear how to best evaluate potential ARI changes in response to interventions in humans. Studies to prove the efficacy of putative longevity drugs in aging people would take decades and cost hundreds of millions of dollars. While strategies are being discussed, and in some cases, deployed toward geroscience clinical trials and biomarker development in humans, so far these are almost exclusively small studies aimed at exploratory endpoints or establishing safety.

One approach toward bridging this gap is to validate longevity interventions from mice in non-human primates. Parallel studies in rhesus macaques were performed between 1987 and 2017 at the University of Wisconsin and at the NIA. However, these did not definitively answer their research question: “Does CR slow aging in non-human primates?” (35, 140). Differences in study design and diet seem likely to account for the differing outcomes (36). These design differences include whether or not to treat midlife and late-life conditions that would be treated in humans, differences in dietary formulation that led to inter-study disparities in the body weights of control animals, and how to adjust analyses for the high incidence of potentially lethal endometriosis—an illness common among virgin female macaques in laboratory settings but not in wild females, for whom pregnancy and nursing lower the risk of this disease. In our view, the development of drugs that could slow aging in humans should not depend on knowing which laboratory protocols are required to document the effect of CR on primate lifespan. Nonetheless, analyses of plasma and tissue changes induced in primates (and in humans) by low-calorie diets may provide very useful indices of a hypothetical “slow-aging” state in primates and humans.

The non-human primate repertoire for aging studies has recently been expanded to include the common marmoset (141). Marmosets have the advantages of being smaller (<1 kg), shorter-lived, and easier to breed than other primates, and they exhibit many of the same pathological features of aging as other primates, including humans. Importantly, marmosets can easily be maintained in extended family groups, consistent with their natural social organization in the wild (141, 142). This is particularly relevant in that social stress due to social isolation or turmoil is well known to impair the health of highly social primates (143, 144). Short-term tests of oral rapamycin in marmosets detected no serious side effects and showed elevation of certain proteostasis markers in some tissues but not others (145–149).

Companion dogs provide another powerful, yet underutilized, model for aging research (150). Unlike laboratory animals, companion dogs share many features of the human environment, living side by side with human owners in a shared household. Companion dogs also participate in a sophisticated veterinary healthcare system that often rivals optimal human healthcare. This sophisticated, preexisting infrastructure for routine care and clinical studies positions companion dogs to make unique contributions to translational aging research.

Dogs age at a rate approximately comparable to that of marmosets and more rapidly than humans. While not a precise calculation, the heuristic of 7 dog years to 1 human year reflects this accelerated rate of physiological decline and biological aging. Similar to humans, dogs experience multisystem functional decline with aging, including deterioration in cardiac, renal, musculoskeletal, cognitive, and immune function (150–152). Age-associated diseases, such as cancer, osteoarthritis, cognitive dysfunction, kidney disease, and heart disease, are prevalent in older dogs, mirroring human geriatric pathologies (152). Lifespan varies dramatically with body size, with larger dogs generally exhibiting shorter lifespans and a more rapid onset of many age-related phenotypes (153). Mixed-breed dogs also tend to live longer than purebred dogs, presumably due to reduced inbreeding and a decreased incidence of breed-specific disorders (154). These patterns make it possible to explore the genetic and physiological determinants of aging rates within the species.

The Dog Aging Project was established in 2014 to aid understanding of, and to study intervention in, the biological aging process of companion dogs (155). This large longitudinal study captures detailed owner-reported data on each dog’s cognitive function, health history, current health status, and environmental conditions, including diet, exercise, sleep, the presence of other animals or humans in the household, and other exposures, such as air and water quality (156). Owner-reported information is cross-compared with veterinary records when possible. Biosamples obtained from a subset of dogs in the longitudinal study are used for various analyses that support translational and precision geroscience, including whole-genome sequencing, blood metabolomics, blood epigenetics, fecal microbiome analysis, and multi-omic analyses (157). All data are made openly accessible to the scientific and non-profit communities, with curated access to biosamples. As of June 2025, data for more than 50,000 unique companion dogs have been obtained.

In parallel with the Dog Aging Project’s longitudinal study, randomized, placebo-controlled veterinary clinical trials have tested whether rapamycin increases lifespan and improves healthspan metrics in dogs. Two completed safety trials showed no significant side effects over 10 weeks (158) and 6 months (159), respectively. In addition, the first trial found evidence of improvements in age-related cardiac function and activity in dogs receiving rapamycin. The Test of Rapamycin in Aging Dogs (TRIAD) clinical trial is ongoing. It is designed to have 80% power to detect a 9% change in lifespan, with lifespan as the primary endpoint. Secondary endpoints include heart function, cognitive function, neurological function, and age-related disease incidence (160). Based on the early successes in the design and execution of these trials, we have now begun to see biotechnology companies developing and seeking United States Food and Drug Administration (FDA) approval for geroscience therapies for companion animals.

## Anti-aging drugs: regulation, economics, and equity

This broad set of interconnected issues (161, 162) has been comprehensively reviewed elsewhere, with attention to regulation (163, 164), ethics and equity (165–169), and economics (170–173). These are areas where bioscientists do not have uniquely apt insight, but a summary of the key points of discussion falls within our intended scope.

### Regulation

Most countries, including the United States, have elaborate sets of rules and regulations to deal with introduction of new medications, including new drugs intended to prevent or postpone diseases. These rules reflect a complex set of historical contingencies related to scientific evidence, the needs of for-profit drug makers and insurance companies, pressure from patients and their relatives, and political agendas. Special legal categories have been created to permit the sale of bioactive agents as “natural products and dietary supplements,” for which little to no evidence of efficacy is required. Although it is presently not legal to directly claim that such products will slow human aging or postpone age-dependent diseases, marketers have easily been able to give potential customers the impression of effectiveness without incurring legal risks for their companies.

Regulatory considerations must take account of the scenario made plausible by recent anti-aging research, in which data from one or more model organisms (i.e., mice and possibly dogs or non-human primates) show that a specific agent extends lifespan and postpones many age-related problems. How would the scientific community test the idea that this agent slows aging, postpones health problems, and extends lifespan with an acceptable side-effect profile in humans? And how would legal strictures on drug approval and physician action respond to such a situation? These issues are already upon us: there is replicable and convincing evidence for the anti-aging effects of several drugs, at least in mice, including a subset of agents approved by the FDA and others that are available without a prescription (14). Data from rapamycin-treated dogs and marmosets (145, 159, 160) should become available within the next few years, and demand for rapamycin, either through off-label prescriptions or extra-legal procurement (174), is already growing.

A wide range of possible paths could potentially lead to the widespread utilization of putative anti-aging drugs by healthy people:

- The FDA might approve a placebo-controlled clinical trial sufficient in size and duration to show whether or not the agent slows aging and postpones diseases in humans. For nearly a decade, a consortium of scientists has been arguing for the Targeting Aging with Metformin (TAME) trial (175), despite the controversy and uncertainty concerning the beneficial effects of metformin beyond diabetes treatment and prevention (176, 177). Discussions between FDA administrators and TAME trial advocates produced an agreement that a composite endpoint—the absence of any one of several common late-life illnesses—would meet the FDA’s criteria for a clinical trial and the scientists’ criteria for an anti-aging drug. The very substantial cost of the proposed trial, approximately US$50 million, has thus far blocked its initiation, despite appeals for financial support from the United States National Institutes of Health (NIH), charitable foundations, and private donors. The inability to garner financial support for the TAME trial may reflect, in part, the lack of financial incentive for putative benefactors, since metformin is off patent and costs little more than aspirin.
  Such studies confront formidable challenges in addition to their long duration and high financial cost. They are clearly too costly to permit parallel testing of multiple agents or multiple doses of the chosen agent. The scientific consensus on the selection of a given agent is very likely to shift as the initial study progresses. Although metformin appeared to be an ideal choice when the TAME trial was first discussed, a decision made today might instead select another agent, such as a sodium-glucose cotransporter-2 (SGLT2) inhibitor (e.g., canagliflozin or a similar drug). To achieve sufficient statistical power for an endpoint such as death or the onset of a lethal illness, these studies must start with volunteers who are old enough to anticipate major health issues within the next 5–15 years, and their design thus makes the tacit assumption that a drug that slows aging in humans will do so even if started at an age near, or just below, the median survival age. In addition, such a study is unlikely to be repeated in a separate population, namely one that differs from the original group in terms of factors such as nationality, ancestry (e.g., populations of East Asian, European, or African ancestry), access to nutrition and healthcare resources, exposure to transient early- or late-life epidemic diseases, and many others. Studies of CR as a candidate anti-aging intervention in rhesus macaques (34, 36) took over a decade to produce even initial results and led to conclusions that varied from site to site depending on protocol nuances; human studies would likely face similar challenges. The prospects for using traditional methods (i.e., randomized prospective clinical trials) to meet FDA criteria for drug approval are therefore quite discouraging.
- An alternate approach involves decisions, by clinicians, to facilitate patient “off-label” access to drugs approved by the FDA or other local regulatory authorities for specific indications. For example, if a physician is convinced by published evidence that a drug could provide health benefits to healthy adult patients, including slower aging and postponement of age-related diseases, they can legally recommend the drug to patients or respond positively to patient requests for the relevant prescription. However, there are many obstacles to this approach. While some physicians prescribe off-label drug use in the near absence of evidence of benefit and despite evidence of potential harm (178), other physicians are very cautious about off-label prescribing or may feel they lack a sufficient grasp of the experimental evidence to take responsibility for off-label drug usage without FDA approval. Evidence of health benefits in humans would certainly be less compelling for agents that have not undergone full-scale testing in randomized trials, and drug safety data for very long-term use (possibly lifetime use) may be absent or conjectural. Some FDA-approved drugs may have been tested for narrow clinical circumstances and for short treatment intervals, such as use of rapamycin for tumor treatment, but have no record of their safety at any dose for long-term administration. Many of the most promising agents developed in mouse aging trials may not have any prior record showing whether they are safe or effective, at any dose, in humans.
- In the absence of regulatory agency-approved or physician-facilitated access to putative anti-aging medicines, many patients have found alternative ways to acquire agents that they themselves consider to have an acceptable risk/benefit ratio. The specific agents chosen may vary from country to country, reflecting legal obstacles to obtaining specific agents and marketing efforts by vendors. This market does not protect against health risks or misleading health claims from aggressive and unscrupulous vendors, and many customers may be overconfident of their own ability to select beneficial preventive agents in such a marketplace.
- Studies of ARIs and metabolomic patterns are in the very early stages, but if additional research can refine and validate their initial promise, it may become possible to use such indices as surrogate markers for the conversion of normal mice, or humans, to a hypothetical slow-aging state. This could enable parallel tests in humans of multiple candidate anti-aging drugs as an initial step toward selecting a small subset for which clinical trials of health benefits are more likely to be successful. For example, if a 1-year treatment of healthy middle-aged adults using metformin, an SGLT2 inhibitor such as canagliflozin, and low-dose rapamycin shows that only one of these candidates induces changes in plasma metabolites similar to those seen in mice given a lifespan-extending drug, this could support a longer trial of that agent in humans or in humans plus a shorter-lived test species, such as marmosets or dogs.

### Economic effects

Researchers have attempted to estimate the possible effects of validated anti-aging drugs on economic activity and life course trajectory. For instance, one study modeled the economic implications of a 20% reduction in the slope of the increase in mortality with age, estimating the societal value of the resulting increase in quality-adjusted life-years (QALYs) at US$7.1 trillion (in 2010 US$) over 50 years (171). More recently, another study projected both medical savings and increased productivity and prolonged economic activity among healthier older people, concluding that a 1-year increase in healthy life in the United States would be worth up to US$38 trillion and up to US$367 trillion over 10 years (170). The 270-fold difference between the two estimates implies that predicting the economic effects of anti-aging drugs is not a simple task, yet, in both models, the economic benefits of targeting aging far outweigh those associated with curing major diseases, such as cancer or cardiovascular disease.

A biological perspective can contribute to these discussions and starts from the observation that interventions (e.g., drugs, diets, and single-gene mutations) that extend the lifespan of mice do so not at the *cost* of health but as a *consequence* of extended intervals of good health. Although the evidence is still limited, it is consistent: long-lived mice, when evaluated for health problems that arise in the last half of the normal lifespan, typically retain relatively youthful function at ages when control mice are increasingly susceptible to age-dependent illnesses and deficits. Hypothetically, a drug could extend healthy human lifespan by approximately 25% (i.e., to the same extent seen in rapamycin-treated mice, CR mice, or mice with GHR or Snell dwarf mutations) and postpone the age of onset of age-dependent illnesses and deficits and thus the age at which they start to impair physical and mental functioning. This scenario is consistent with mouse data, although the experimental evidence is incomplete in other animals and essentially absent in humans. Taken together, these findings suggest that extending lifespan need not entail prolonged frailty but may instead reflect a delay in the onset of age-dependent illnesses and functional decline—an interpretation that is well supported in mouse models but that is far less well tested in humans.

The economic effects of such a radical advance in preventive medicine are impossible to predict with confidence and may be affected by differential access to the drug(s) used, social pressures and expectations, regional and national variations in resource availability, and the considerable health effects expected due to the accelerating global climate catastrophe. Similar issues would have potentially confronted speculative economists 200 years ago if they had been asked to consider the possible economic impact of antibiotics, anesthetic-assisted surgery, advances in sanitation systems, the discovery of insulin, and the development of vaccines for many serious infectious conditions. A changing population, in which fewer people died in infancy and childhood and a greater percentage survived beyond the age of 30 or even 60, would be a very plausible outcome. The “medical” developments of the past 200 years have surely had a substantial effect on economic networks at all scales, alongside other technological advances (e.g., in transportation, communication, fuel mix, computer networks, engineered food stocks, and more). We suspect that those who confidently project the economic effects of putative anti-aging medicines are overconfident, while those who primarily project ill effects from such research advances should be asked if they would recommend reverting to the medical system of the early 1800s.

### Equity issues

Discussions of the possible effects of validated anti-aging drugs frequently evoke the question of differential accessibility—the idea that such drugs might only be available to the wealthy and thus might exacerbate the current, indefensible, disparities between rich and poor in access to medical care and in measurable health outcomes. These disparities in healthcare access and health outcomes have already arisen in an environment without any authentic anti-aging drugs, and hence the challenge of diminishing the effects of wealth-related disparities is independent of the potential contributions of anti-aging medications to health-maintenance strategies. Some tactics, such as access to medical care, healthy foods, satisfying work, and supportive social structures, are of course already much more easily available to wealthy people and in wealthy countries and have large, quantifiable benefits to lifespan and to disease risk. This problem and its potential solutions are in the main orthogonal to considerations of the ethics of developing anti-aging drugs.

Notably, the majority of leading candidate drugs that have been successful in rodent lifespan tests are not forbiddingly expensive. Many of the most potent and valuable interventions available to modern clinicians, from smallpox and measles vaccinations to fluoridated water to antibiotics to vitamin supplements, can be produced and distributed at affordable costs to nearly all residents of high-income countries, and low- and middle-income countries. The high costs of novel drugs often reflect a complex set of factors related to commercial profit-seeking and regulatory protections, rather than to high marginal production costs. If novel anti-aging agents are eventually discovered and validated, they would not necessarily be so costly to produce as to exacerbate current inequities in access to medical care. Indeed, if they postpone or prevent common, costly conditions, anti-aging drugs could well redress some of the inequities in access to modern medical care.

## Controversial topics in anti-aging research

The field of anti-aging faces various controversies important for its future direction. This section addresses the topics of hallmarks of aging, healthspan, cellular senescence, “aging clocks”, and “gerontologiphobia”—many of which have implications for the ARI research roadmap.

### Hallmarks of aging

The “hallmarks of aging” rubric, introduced in 2013 and updated in 2023 (179, 180), proposed a set of interconnected biological processes that characterize aging across species. This framework provided a unifying language for a field that had historically lacked one and allowed scientists and popular communicators to explain aging to broader non-specialist and public audiences. These hallmarks have also had an outsized influence on the direction of aging research and commercialization by guiding which lines of investigation are prioritized and which are not pursued through indirect effects on resource allocation. This reliance on a list of hallmarks is a “double-edged sword” that may have hindered the field as much as it has helped.

The tabulation of nominal hallmarks requires and reflects professional opinion and is therefore the outcome of an arbitrary and subjective procedure. Hallmarks are not natural constructs, such as the periodic table or a genetic sequence. They are a human-made construct that imperfectly represents what we—or rather, the authors of the two cited articles (179, 180)—think we know about biological aging today. Tables of hallmarks of aging are likely to be less stable and less reliable than models of electron transport, immune cell categorization, or atheroma formation, because we still know relatively little about the underlying complexity of biological aging compared with more mature fields, such as cancer and cardiovascular research.

A second problem is causality. One criterion for inclusion as a “hallmark” is that modifying the hallmark should delay aging. But demonstrating this causality has been challenging in practice. Many of the hallmarks undoubtedly *correlate with* age and age-related decline, but evidence of a causal association is often lacking. Similar critiques have been raised against “hallmarks” in other fields, such as cancer (181), where the boundaries between core processes and downstream effects often blur.

A third limitation relates to the interaction among hallmarks. While both the 2013 and 2023 studies (179, 180) noted that understanding the interaction among the hallmarks was critical, the model itself tends to imply that each hallmark is a discrete, independently targetable module—an oversimplification known to be seriously flawed. Multicellular organisms, from worms and flies to mice and humans, are finely tuned, networked systems in which changes in one biochemical pathway, cell, tissue, or system typically ripple through many others. Loss of proteostasis can affect mitochondrial function, which in turn influences nutrient sensing and cellular senescence. This systemic interconnectedness may be the reason why some interventions, such as rapamycin, canagliflozin, and CR, can affect so many age-related phenotypes simultaneously. The hallmarks framework does not help us model or predict these cross-talk dynamics. If anything, it risks reinforcing a modular view of aging that does not reflect biological reality.

There is a particularly problematic consequence of reifying tabulated hallmarks as a map of what is worth studying in biogerontology: a shift of resources away from lines of investigation that are either exploratory or that do not fit neatly into the hallmarks framework. Evolving models of how aging leads to decline, and studies of factors that can slow aging rates, are almost certainly incomplete, and further progress is likely to depend on new concepts and discoveries not yet enshrined in the hallmark pantheon. Exploratory science, including both hypothesis-based studies and exploratory screens of genes, RNA levels, proteins, and metabolites, is certain to yield valuable insights into cellular structures and circuits that were not considered critical in 2013 or 2023. The hallmarks framework conveys a false confidence that we already understand the biology of aging and the links between aging and late-life decline. To the extent that popularization and high-level endorsement of the hallmarks framework set constraints for “good aging science,” the adoption of this unwarranted filter can make it increasingly difficult to obtain funding for discovery approaches in the field.

### Healthspan versus lifespan

Ever since scientists began investigating the biology of aging with the goal of discovering interventions to make us live longer, there have been concerns about the quality of that extended life. Would we be extending healthy life or only the period of debility that the majority of people suffer near life’s end? The term “healthspan” was coined to emphasize that the scientific goal was to extend healthy life, not unhealthy life (182). Looking at the recent history of human health and longevity, this distinction seems legitimately important. For instance, health epidemiologists evaluating healthy life expectancy (HALE)—a metric based on activity limitations due to more than 300 diseases and injuries—found that between 1990 and 2016 in the United States, life expectancy increased by 4.5 years in men and 2.4 years in women, but approximately one-quarter of the increase in men and one-third of the increase in women consisted of additional years lived in poor health (183). This pattern is not surprising, given that our modern healthcare system is focused on preventing death rather than preventing disease and debility. To further illustrate why the gap between healthy life and total life has been increasing, it should be considered that, in the United States, during the first 19 years of the 21st century (excluding the confounding impact of the COVID-19 pandemic), age-adjusted rates of death from heart disease and cancer—the two leading causes of mortality—fell by 37.4% and 27.3%, respectively, but life expectancy increased by a mere 1.9 years (184, 185). Because of medical progress, people who might have previously died now survive their heart attack or cancer for additional years, but they often live those years in compromised health due either to disease sequelae or to the treatments that saved them (186). By directly targeting the biology of aging, as geroscientists routinely do in model organisms, later-life diseases and debilities can be delayed or prevented simultaneously, thus extending healthy life. Although there have been reports that specific interventions extend health without extending life (187, 188), we are unaware of any examples in mammals in which life extension has not been accompanied by health extension. Whether the period of ill health toward the end of life could also be extended in laboratory rodent studies is a question not answerable by current studies, because animals in palpably ill health are, by regulation, humanely euthanized to mitigate their suffering (189). In *C. elegans*, there is evidence that at least one genetic intervention that extends life does lengthen the period of debility at the end of life, but this appears to be due to a worm-specific phenomenon caused by an overabundance of live bacterial food (190, 191). Since there are no current interventions proven to slow aging in humans, discussions about the extent to which anti-aging drugs might increase the absolute or relative amount of healthy human life are at present speculative. Detailed studies of age-dependent function, such as changes in bone, immunity, cognitive function, muscle strength, and many other outcomes pertinent to human well-being, could, however, be incorporated into ongoing and future studies of anti-aging drugs in mice to provide empirical evidence to inform and constrain speculation about the likely effects of hypothetical anti-aging drugs on human healthspan.

### Cellular senescence

The observation (192) that human diploid fibroblasts typically cease dividing after approximately 50 population doublings in a Petri dish was rapidly adopted by many in the cell biology community as an *in vitro* model of “the aging process”, that is, the complex, systemic progression from youthful health to age-associated decline and disease. This interpretation was embraced despite the limited justification for equating mitotic arrest in fetal fibroblasts with the diverse set of changes observed in aging organisms. Aging *in vivo* involves functional changes in both mitotic and post-mitotic cells, alterations in extracellular matrix composition, dysregulation of endocrine and immune feedback systems, and shifts in stem cell activity, among many other processes. Against this backdrop, it is difficult to justify the assertion that growth cessation in a homogeneous culture of fetal lung fibroblasts meaningfully represents the aging of an intact multicellular organism. Nonetheless, the advent of techniques for culturing non-transformed cells *in vitro* understandably attracted researchers, many of whom were enthusiastic about using these new experimental tools, although the model’s relevance to organismal aging remained largely unexamined.

The discovery that growth arrest in cultured cells correlates with telomere shortening prompted a second wave of interest (193, 194). This led to the hypothesis that telomere attrition is a central driver of aging, despite considerable species differences in telomere biology. The adoption of the term “cellular senescence” to describe this late-passage growth arrest reified and reinforced a conceptual framework in which aging is considered to be primarily a cell-autonomous process, potentially driven by cumulative mitotic divisions. This idea has influenced two generations of researchers and shaped much of the contemporary discourse around aging. Referring to *in vitro* telomere-dependent growth cessation as “cellular senescence” continues to serve as a powerful lure in the aging research community, despite Alfred Korzybski’s famous warning (195) that “the map is not the territory” and “the word is not the thing”.

A number of substantive objections challenge the centrality of replicative senescence and telomere shortening as general mechanisms of aging. First, age-associated dysfunction is evident in both short-telomere species (such as humans) and long-telomere species (such as mice and dogs), raising questions about the universality of telomere-driven mechanisms. The spectrum of age-dependent changes is very similar in mice, dogs, and humans, and it is implausible that these deficits are produced by telomere shortening in one species but by an unrelated process in another. Second, many human tissues, such as the skin and the gut, undergo continuous cell renewal throughout life without evidence of replicative exhaustion. Third, aging-related decline occurs in largely post-mitotic tissues such as the brain and heart, where telomere dynamics and replicative exhaustion can play little direct role. Fourth, human disorders of telomere maintenance, such as dyskeratosis congenita, produce clinical syndromes that are quite distinct from normative aging. Finally, telomerase-deficient mice exhibit no overt aging phenotypes for several generations, and when dysfunction does eventually appear, at the sixth generation, it differs markedly from typical aging-related pathologies (196).

Over the past two decades, research on “cellular senescence” has expanded beyond the original framework. Researchers pursuing the idea that cellular senescence is a key causal factor in mammalian aging now believe that there are many ways, other than sequential mitosis, to induce a cell to turn senescent, including DNA damage, oxidative stress, oncogene activation, and more (197, 198). Leaders in this field have also turned their attention to other properties of senescent cells, such as their resistance to apoptosis and the production of cytokines and inflammatory mediators, collectively referred to as the senescence-associated secretory phenotype, or SASP (199). Finally, the set of cellular phenotypes considered to be diagnostic of senescent cells has greatly expanded to include changes in cell cycle regulators, the production of variable sets of secreted proteins, and changes in lysosomal properties (200–202). The central core of this conceptual system is that aging is something that can happen to cells, that cells can acquire an “aged” phenotype, and that this transition contributes causally to organismal aging. This framework has fueled interest in senolytic drugs, compounds designed to selectively eliminate senescent cells, and in rejuvenation strategies aimed at reverting cells to a more youthful state (203, 204). While this line of inquiry is active and well-resourced, evidence of its efficacy in improving healthspan or extending lifespan remains limited. For example, a recent ITP study testing the purported senolytic agent fisetin found no impact on lifespan and no evidence of senescent cell clearance in any of three tissues using a panel of biomarkers considered suitable for the detection of senescent cells (14).

The growing complexity and variability of senescence-associated markers, along with inconsistent findings when this toolkit is applied to various cell types and different modes of senescent cell production, has prompted some researchers in the field to question whether a singular “senescent cell” type exists. The emerging consensus is that there are multiple types of senescence (205). A more nuanced paradigm is emerging that recognizes diverse, cell-type-specific, age-associated changes. Rather than presuming a common senescence state, this perspective highlights heterogeneity: neural stem cells may lose differentiative ability; glial cells may alter their cytokine profiles (206); and pancreatic β cells may show cell-cycle arrest in the context of metabolic stress (207). These changes may or may not involve subsets of the canonical features of senescence as defined in fibroblast models. This broader framework invites a wide range of potentially productive research questions. For instance: what factors (genetic, toxic, hormonal, or nutritional) can produce age-dependent changes in inflammatory mediators in various glial cells in different areas of the brain? How do specific forms of age-dependent cellular changes in the immune system alter the speed and direction of cell expansion after an infectious challenge? Do age-dependent changes in the number, responsiveness, and properties of multipotent “stem cells” in muscle, the endocrine pancreas, skin, the brain, and adipose tissue contribute to late-life loss of function in these tissues and in the cells that they influence throughout the body? A conceptual model that focuses too narrowly on eliminating a single, poorly defined class of “senescent cells” may miss the opportunity to explore these fundamental questions. Recognizing the diversity of age-related cellular phenotypes rather than lumping them under a single, contested label will ultimately yield deeper insights into the biology of aging and point toward more effective therapeutic interventions.

### Marketing of aging clocks and purported anti-aging products

The marketing of products that purport to measure or slow biological aging is a significant challenge facing the anti-aging field. From a scientifically rigorous perspective, neither goal has been accomplished, and such marketing claims are, by definition, dishonest. However, because of the lack of effective laws or regulatory enforcement to prevent such behavior, aging tests and anti-aging supplements are ubiquitous in the direct-to-consumer market. In some cases, the problem is exacerbated by academic scientists who allow themselves to be associated with these products or even endorse them directly, leading to confusion among consumers who may believe that these products have been shown to work.

“Aging clocks” are algorithms that identify a correlation between a finite set of biomarkers (molecular, physiological, or both) and an age-correlated phenotype. The age-correlated phenotype often includes one or more of the following: chronological age, future mortality risk (e.g., 3-year, 5-year, 10-year), or a specific age-related disease incidence risk (e.g., specific types of cancer, cardiovascular disease, kidney disease, or dementia). So-called “aging-rate clocks” similarly identify a correlation, but in this case the biomarkers are correlated with the rate of change in an age-dependent phenotype, such as the rate of change in a composite frailty or health index. Importantly, despite the fact that these algorithms are marketed as “biological aging clocks” or “pace of aging clocks,” they were not developed to directly correlate with biological age or biological aging rate. Indeed, that would be impossible, as currently we have no way to measure biological age or biological aging rate. Thus, all “aging clocks” are, at best, associated with something that correlates with biological aging. A recent review has outlined some of the conceptual and empirical problems facing advocates of molecular “clocks” as surrogate endpoints for anti-aging intervention studies (208).

The most common “aging clocks” estimate biological age or (less often) biological aging rate using a subset of DNA methylation sites that have been shown to correlate with one or more of the age-correlated features described above. These algorithms are generally developed in academic research based on large longitudinal datasets from human or animal studies. Numerous companies have taken these algorithms and developed consumer products that claim to accurately measure biological age. At present, none of these consumer tests have been validated by any independent body or received any certification or FDA approval. Consumers are tempted to believe that these tests have been proven effective and that they accurately measure a valuable health metric. Some physicians are even recommending that their patients take these tests, despite the lack of information related to precision or accuracy and the absence of actionable implications even if the tests are accurate in some illdefined sense (209, 210). Companies that bring such tests to market have incentives to do so with little to no transparency regarding quality control, accuracy, precision, or utility, and there is limited regulatory oversight regarding these features or claims about measuring “biological aging”.

Similarly, the nutritional supplement market has long been problematic in the context of “anti-aging therapies.” While supplement manufacturers are expected to meet regulatory requirements concerning ingredient quality and label accuracy, the FDA has, in general, given broad leeway for claims about hypothetical efficacy. Manufacturers who make outright false claims can be prosecuted but generally are not, and overtly misleading claims about efficacy are rampant (210, 211). A particular challenge is the rush to market of supplements based on a single study, often in an invertebrate animal model reporting lifespan or healthspan benefits. As long as the compound in question has the designation of “generally recognized as safe”, there is minimal commercial downside to marketing the product as a “longevity” or “anti-aging” supplement for human consumption. In such a regulatory environment, companies are disincentivized to test their products for efficacy, hesitating to spend money on studies that show no clinical benefits.

### Gerontologiphobia

Among the many obstacles slowing the development of clinically accepted anti-aging drugs, perhaps the most obstructive is “gerontologiphobia,” the irrational belief that while research on diseases that afflict the aged is honorable and meritorious, research aimed at slowing aging itself—and thus all late-life illnesses—should be condemned and discouraged (162, 212–214). Gerontologiphobia, a neologism defined as the fear of knowledge about aging, has several variants, including a fear that anti-aging drugs would create a large population of ill, unhappy people, possibly living with dementia, who wish they could die but are kept from doing so by medical science; and the contradictory fear that keeping people alive, healthy, and productive for a long time would lead to ill-specified economic debacles. Logically, these same concerns would prompt the termination of research into the prevention and treatment of common lethal diseases (e.g., heart attacks and cancer), whose goal, similar to that of applied biogerontology, is to help people postpone disease-related declines and death. We admire the passion and creativity of our colleagues working within the “one disease at a time” framework that consumes >99% of NIH research support. However, demographic calculations (215, 216) estimate that the complete elimination of all forms of neoplasia after the age of 50—an outcome that appears to be unlikely to occur any time soon—would increase the median lifespan of people in a wealthy modern society by a mere 2–3%. This is approximately 10 times smaller proportionally than the increase in median lifespan already produced, reproducibly, in mice by the combination of rapamycin plus acarbose in ITP studies (61).

Barriers to the development of anti-aging drugs with documented clinical efficacy have remained largely unchanged for over 20 years (212). These include the stigmatization of the field caused by prominent promoters of ineffective nostrums, a lack of commercial interest within for-profit companies, which typically seek the rapid development of remedies for specific diseases; passionate advocacy for disease-specific research by people affected by these conditions (including patients, relatives, and friends); and the still common, although now firmly refuted, conviction that the aging process cannot be slowed. The conviction that success in anti-aging research would be harmful, while indefensible and easily refuted, is still widely held (214, 217), and poses a major problem for progress in this field. Nearly all people are eager to take steps (such as exercising, wearing seatbelts, taking antihypertensive medications, or undergoing surgery to remove accessible neoplasms) to increase their chances of staying alive, healthy, and active for another 3–20 years, and the majority of people would be happy to take a medication that could produce the same protection from the multiple consequences of aging (217). The intuition that such a hypothetical anti-aging medication, while ethically acceptable for oneself, would be unethical for others to access does not hold up to scrutiny.

## Conclusions

Over the past few decades, research into the biology of aging has catalyzed a significant paradigm shift: aging in mammals is not immutable. It can be modulated not only through CR or rare genetic mutations but also by pharmacological interventions administered during adulthood. Each new example of an effective anti-aging drug provides specific molecular and physiological clues to the control of the rate of aging and the search for anti-aging drugs. Each new example also contributes a tool toward the goal of identifying shared changes that can point the way to so-far mysterious upstream control pathways, potentially manipulable by interventions. The surprising finding that effective anti-aging drugs are often fully active when initiated in late adulthood suggests that similar preventive anti-aging medicines might be effective when administered to middle-aged people. ARIs, as measurable outcomes that can discriminate normal mice from slow-aging mice even in early adulthood, have the potential to serve as a bridge to more rapid screening of candidate anti-aging drugs both in rodent studies and in human clinical trials of relatively short duration.

It is time to shift our focus from the quest to define some hypothetical single “cause” of aging to a more actionable and evidence-driven question: what determines variation in the rate of aging within a species? Growing scientific and public recognition of the legitimacy and promise of this approach could help reduce gerontologiphobia and elevate aging biology to a level of urgency and respect comparable to research into the causes and treatment of cancer, diabetes, atherosclerosis, dementia, and the myriad other slings and arrows that aging flesh is heir to.

## Figures and Tables

**FIGURE 1:**
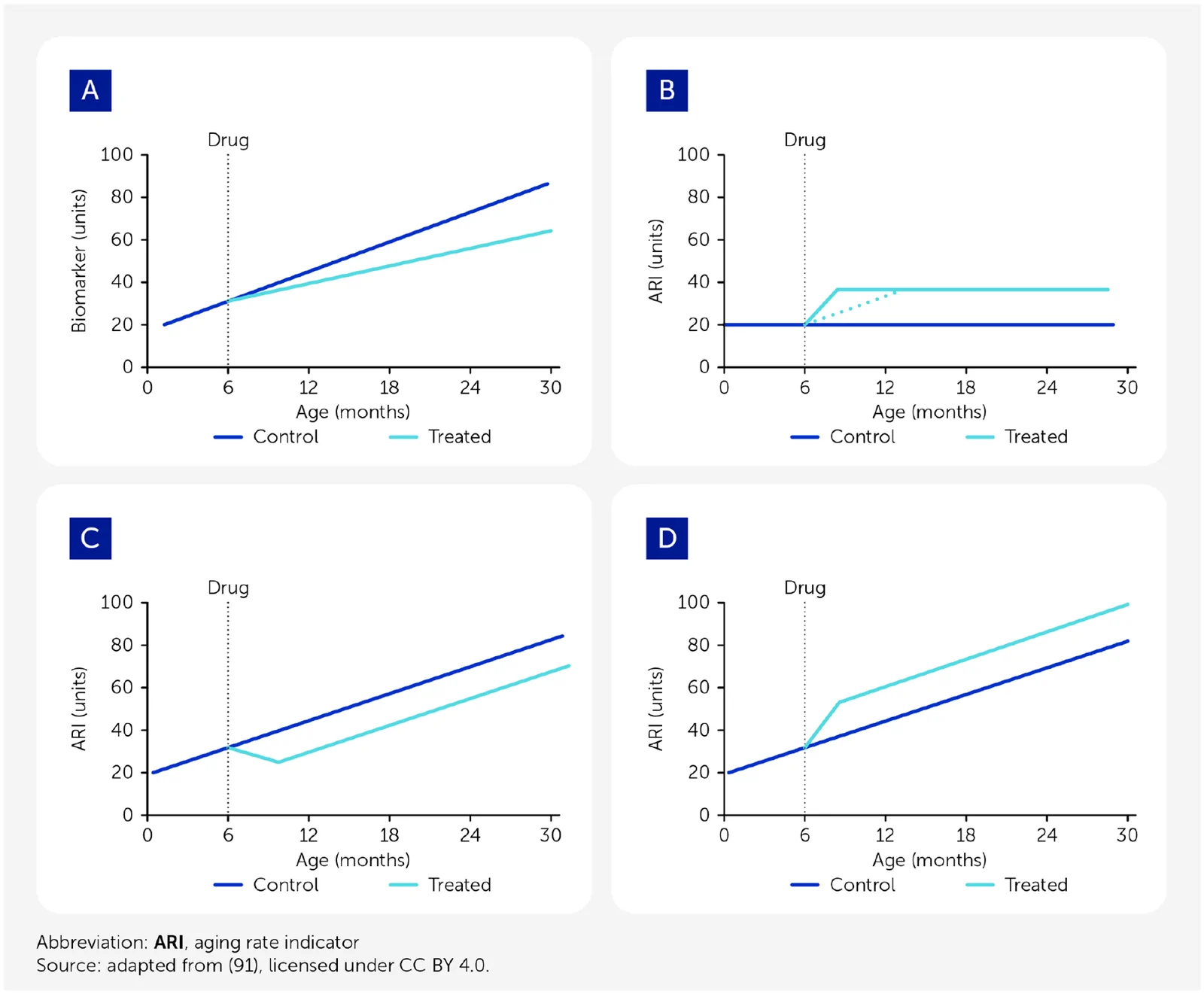
Hypothetical comparison of a biomarker of aging to several aging rate indicators (ARIs) in the context of a response to an anti-aging drug. **(A)** The biomarker shows a steady age-dependent increase from 2 to 30 months of age (dark-blue line), and the rate of increase is lower in drug-treated mice (light-blue line). The difference between control and treated mice becomes easier to detect; that is, it is detectable with smaller group sizes, only at older ages. **(B)** The hypothetical ARI illustrated does not change with age in control mice, and drug treatment (initiated at 6 months) causes a change in ARI level until a higher, steady state is reached. The actual rate of change between normal and slow-aging states is not yet well documented for any ARI, although ARI effects are clear by 6 months of age in mutant mice and are established between 4 and 12 months in drug-treated and calorie-restricted (CR) mice. The dotted line segment illustrates a more gradual change than the solid line segment. The relatively rapid effects of drugs and a CR diet on ARIs make it possible to detect the effects of an intervention in a comparatively short timeframe and in younger mice. In other situations, an ARI might increase with age **(C, D)**, and the intervention could cause the ARI to decline **(C)** or increase **(D)** within a few months. It is equally possible for biomarkers or ARIs to diminish in older mice (not illustrated). Adapted from (91), licensed under CC BY 4.0.

**FIGURE 2:**
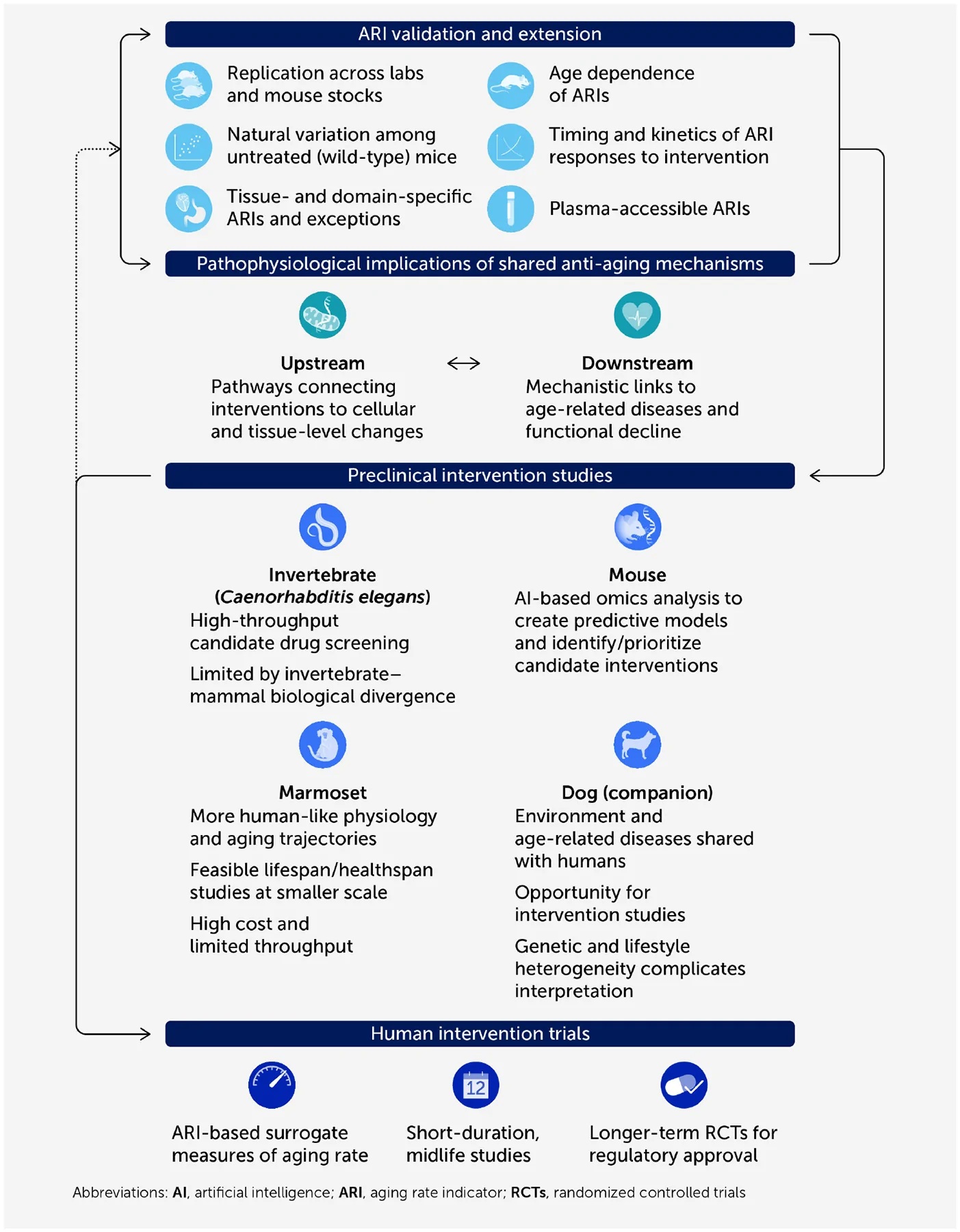
Research roadmap for the further development and validation of aging rate indicators and their application in the research and development of anti-aging interventions (drugs and/or diets).

**FIGURE 3:**
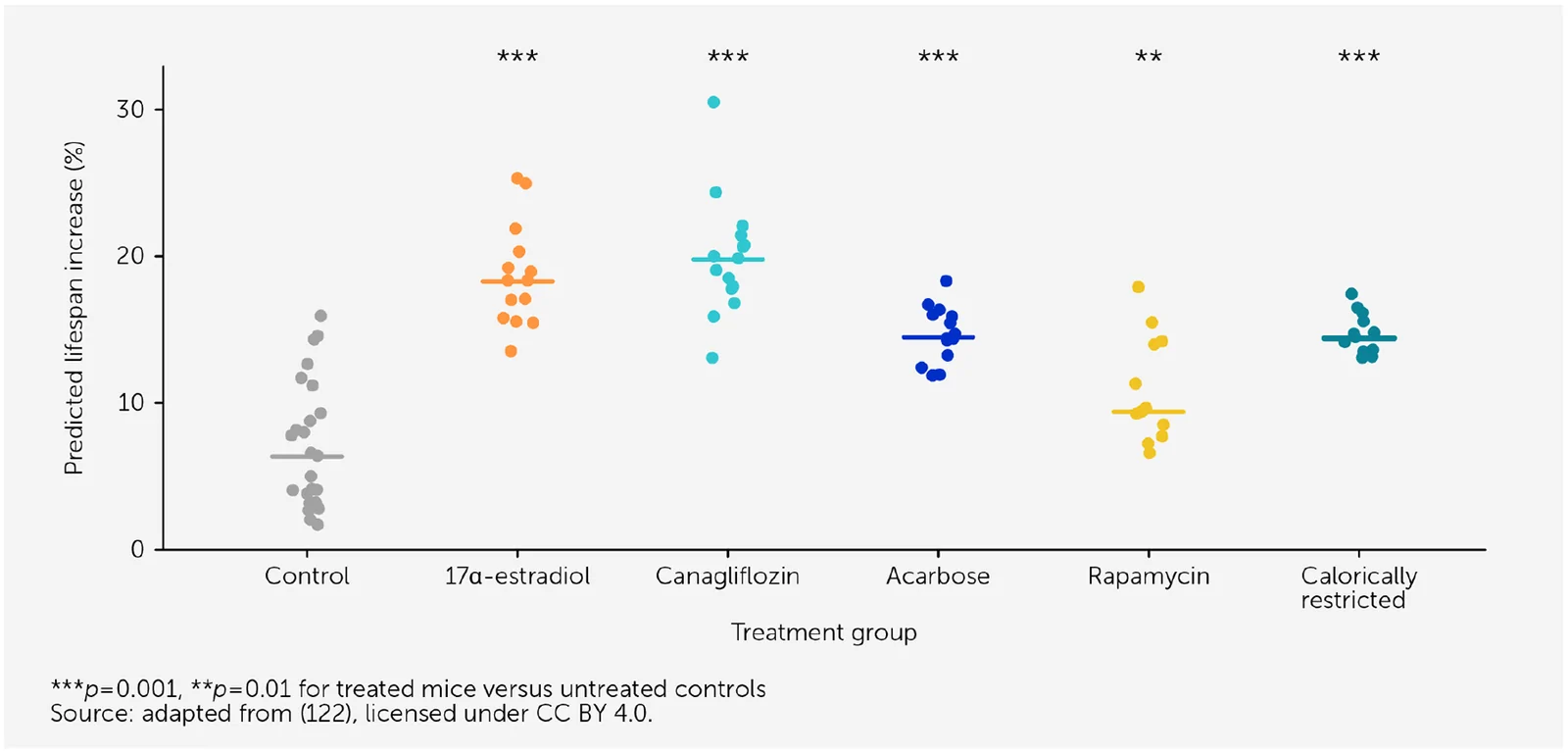
Plasma metabolite profiles from 1,051 identified metabolites were used to train XGBoost regression models for male UM-HET3 mice at 12 months of age. In each case, the model was trained using data from untreated mice plus four varieties of slow-aging mice, and then it was used to predict the “estimated lifespan increase” (Y-axis) based on the metabolite levels of each mouse in the fifth treatment group. Drug treatment or calorie restriction was initiated at 4 months of age. Each symbol represents one mouse, and the horizontal bars show the group means. Values are based on a series of 1,000 independent models, averaged for each mouse. Asterisks indicate that each group of treated mice had a significantly higher level of predicted lifespan increase than the untreated control group (***p=0.001, **p=0.01). Adapted from (122), licensed under CC BY 4.0.

**FIGURE 4:**
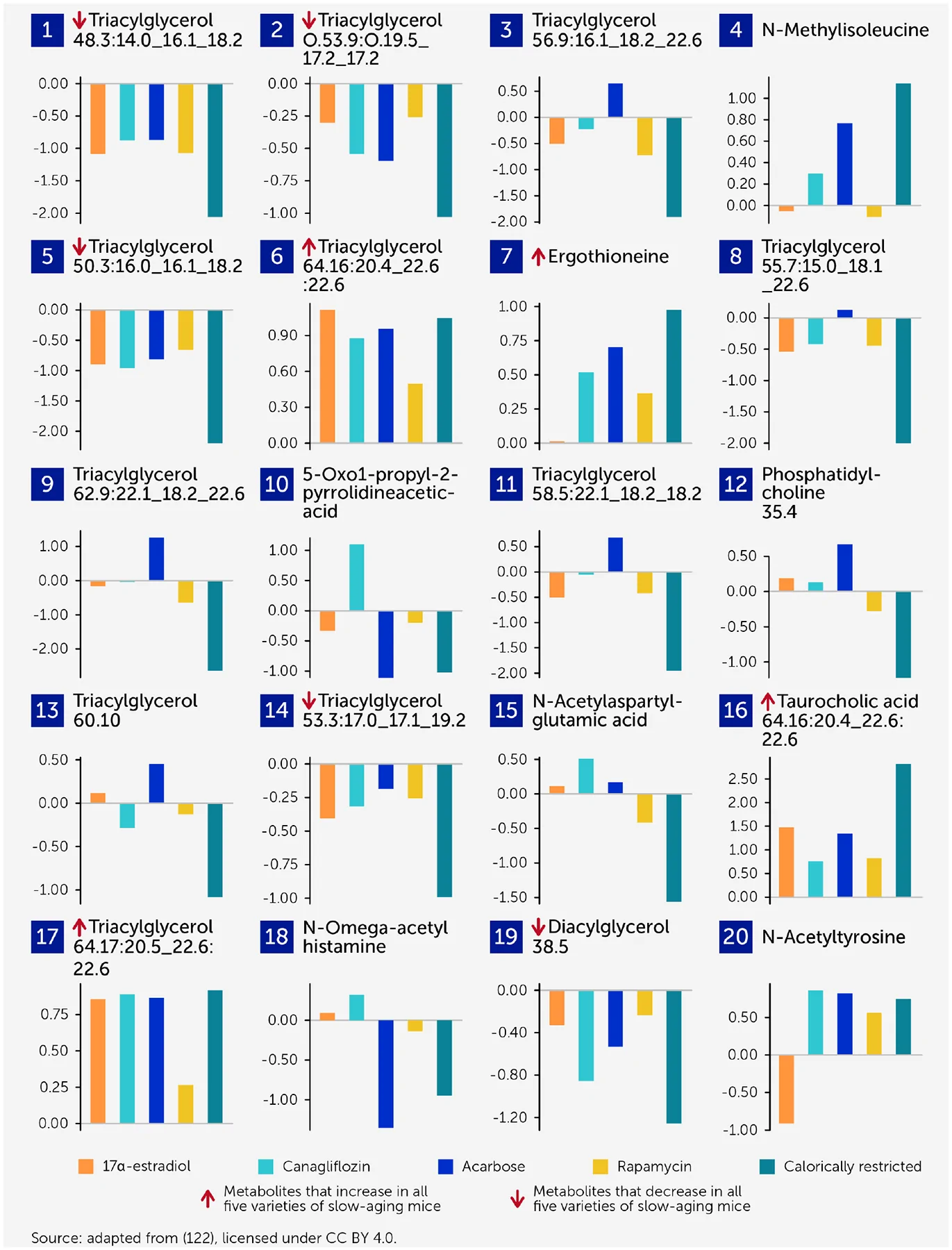
Bar plots showing average log_2_ fold changes for each of the 20 plasma metabolites with the largest influence on predicted lifespan increase values for male UM-HET3 mice using the “novel intervention test” depicted in Figure 3. Metabolites are ranked by the size of the “gain,” which reflects feature importance and was estimated from the trained XGBoost regression models for male mice. Upward arrows indicate metabolites that increase in all five varieties of slow-aging mice and downward arrows indicate metabolites that decrease in each slow-aging model. (Note differing Y-axis scales.) The method is similar to that used in Badenoch et al. (122), except that relative feature importance values in that study have been replaced by the native “gain” metric. Values shown represent the median values observed in 1,000 computer runs, each of which was initiated with a different random seed. Adapted from (122), licensed under CC BY 4.0.

**TABLE 1:** Agents or combinations that have produced a statistically significant increase in the lifespan of mice in one or both sexes in studies conducted under the auspices of the United States National Institute on Aging (NIA)-supported mouse Interventions Testing Program (ITP).

Drug(s)	Effects (at best dose)	Max	Rep	Notes
Rapamycin	Males 23%, females 26%	Yes	Yes	Late start: ~100% (9, 58, 59)
Acarbose	Males 22%, females 5%	Yes	Yes	Late start: ~50% (10, 13, 60)
Rapamycin + acarbose	Males 29%, females 22%	Yes		UT omitted (see study) (61)
17α-estradiol	Males 19%	Yes	Yes	Late start: ~100% (10, 60, 62)
Canagliflozin	Males 14%	Yes	Yes	Late start: ~100% in males, toxic to females (11, 63, 64)
NDGA	Males 12%		Yes	(10, 65)
Glycine	<5%, both sexes	Yes		(66)
Astaxanthin	Males 12%		Fail	(14)
Meclizine	Males 8%		Fail	(14)
Protandim	Males 7%			(60)
Captopril	Females 4%			UT omitted (see study) (61)
16-hydroxyestradiol	Males 15%	Yes		Toxic to females (63)
Mitoglitazone (MSDC-160)	Males 9%			(67)
Halofuginone	Males 9%	Yes		(67)
Epicatechin	Males 5%	Yes		(67)

Max = a surrogate for “maximal lifespan”, i.e., does the agent or combination have a significant effect on the probability of survival to the 90th percentile of the joint survival distribution? Rep = “replicated”, i.e., it produced a significant survival advantage, as measured by log-rank *p*<0.05, in at least two distinct cohorts initiated in two different years.

Abbreviations: **NDGA**, nordihydroguaiaretic acid; **UT**, University of Texas.

**TABLE 2:** Candidate aging rate indicators: shared mechanisms found in slow-aging mice in studies conducted under the standardized protocols of the United States National Institute on Aging (NIA)-supported mouse Interventions Testing Program (ITP).

Endpoint	Tissue(s)	Seen in these models (* indicates males only; F = females only)
CIT	Liver, kidney, muscle	Snell, Ames, GHRKO, PAPP-A KO; Ile-R; Rapa, Aca, 17aE2*
MEK1/ERK	Liver, kidney	Ile-R; Rapa, Aca, 17aE2*, Cana*
MEK3/p38	Liver, kidney	Rapa, Aca, 17aE2, Cana
mTORC1	Liver, heart, kidney, muscle	Snell, GHRKO, PAPP-A KO; CR; Ile-R; Rapa, Aca, 17aE2*, Cana*
UCP1	BAT	Snell, Ames, GHRKO, PAPP-A KO; CR; Aca, 17aE2, Cana; not Rapa
UCP1	Ing WAT	Snell, Ames, GHRKO, PAPP-A KO; CR; Aca, 17aE2, Cana; not Rapa
UCP1	PG WAT	Snell, Ames, GHRKO, PAPP-A KO; CR; Aca, 17aE2, Cana; not Rapa
M1 and M2	BAT, Ing WAT, PG WAT	Snell, Ames, GHRKO, PAPP-A KO; CR; Rapa, Aca, 17aE2, Cana
FNDC5	Muscle	Snell, Ames, GHRKO, PAPP-A KO; CR; Rapa, Aca, 17aE2*, Cana*
Irisin	Plasma	Snell, GHRKO, PAPP-A KO; CR; Rapa, Aca, 17aE2, Cana
GPLD1	Liver, plasma	Snell, Ames, GHRKO, PAPP-A KO; CR; Rapa, Aca, 17aE2, Cana
BDNF	Hippocampus	Snell, Ames, GHRKO, PAPP-A KO; CR; Rapa, Aca, 17aE2, Cana*
DCX	Hippocampus	Snell, Ames, GHRKO, PAPP-A KO; CR; Rapa, Aca, 17aE2, Cana
p65 (NF-κB)	Liver	Snell, GHRKO, PAPP-A KO (F); CR; Rapa, Aca, Cana; not 17aE2

Abbreviations: **17aE2**, 17α-estradiol; **Aca**, acarbose; **BAT**, brown adipose tissue; **BDNF**, brain-derived neurotrophic factor; **Cana**, canagliflozin; **CIT**, cap-independent translation; **CR**, calorie restricted; **DCX**, doublecortin; **ERK**, extracellular signal-regulated kinase; **FNDC5**, fibronectin type III domain-containing protein 5; **GHRKO**, growth hormone receptor knockout; **GPLD1**, glycosylphosphatidylinositol-specific phospholipase D1; **Ile-R**, isoleucine-restricted diet; **Ing WAT**, inguinal WAT; **M1/M2**, pro-inflammatory and anti-inflammatory macrophage phenotypes, respectively; **MEK**, mitogen-activated protein kinase kinase; **mTORC1**, mammalian target of rapamycin complex 1; **NF-κB**, nuclear factor kappa-light-chain-enhancer of activated B cells; **p38**, p38 mitogen-activated protein kinase kinase; **p65**, NF-κB p65/RelA subunit; **PAPP-A KO**, pregnancy-associated plasma protein A knockout; **PG WAT**, periogonadal WAT; **Rapa**, rapamycin; **UCP1**, uncoupling protein 1; **WAT**, white adipose tissue.

Sources: (3, 57, 92–101).

Adapted from (91), licensed under CC BY 4.0.

## Data Availability

Publicly available datasets were analyzed in this study. Specifically, this data was first presented and analyzed in (122). It can be found here: https://zenodo.org/records/17780311.

## Key points

- Agents or combinations shown to extend mouse lifespan by 12% or more, in one or both sexes, include rapamycin, acarbose, rapamycin plus acarbose, 17α-estradiol, 16-hydroxyestradiol, canagliflozin, nordihydroguaiaretic acid (NDGA), and astaxanthin.
- Physiological and molecular changes proposed as candidate aging rate indicators (ARIs) include levels of uncoupling protein 1 (UCP1) in adipose tissue, glycosylphosphatidylinositol-specific phospholipase D1 (GPLD1) in the liver, the ratio of inflammatory to anti-inflammatory macrophages in fat, the activation of cap-independent protein translation, and the down-regulation of mammalian target of rapamycin (mTOR) complex 1 (mTORC1) activity.
- By discriminating normal mice from slow-aging mice (even in early adulthood), aging rate indicators could enable more rapid screening of candidate anti-aging drugs in mice and, potentially, in human clinical trials of relatively short duration.
- It is hoped that the proposed ARIs will help guide research into the pathogenesis of many age-related conditions, such as diabetes, cancer, cardiac disease, and neurodegenerative diseases.
